# Endophytic Fungi from Wild *Polygala crotalarioides*: Diversity, Antioxidant Capacity and Acetylcholinesterase Inhibitory Potential

**DOI:** 10.3390/jof12090669

**Published:** 2026-09-06

**Authors:** Kaize Shen, Mingjian Xu, Xianchun Cai, Man Luo, Hongyin Zhou, Xun Dai, Wei Zhou, Shunqiang Yang

**Affiliations:** 1 Yunnan Key Laboratory of Gastrodia and Fungi Symbiotic Biology, Zhaotong University, Zhaotong 657000, China; ztushenkz@163.com (K.S.); xumingjian0228@163.com (M.X.); v3541989640@163.com (X.C.); 19984053548@163.com (M.L.); zhy1605202632@163.com (H.Z.); 06016@ztu.edu.cn (X.D.); 2 Yunnan Engineering Research Center of Green Planting and Processing of Gastrodia, Zhaotong University, Zhaotong 657000, China; 3 School of Agronomy and Life Sciences, Zhaotong University, Zhaotong 657000, China; 4 Yunnan Key Laboratory of Smart Villages and Agri-Cultural-Tourism Integration, Zhaotong University, Zhaotong 657000, China

**Keywords:** Wild *Polygala crotalarioides*, endophytic fungi, fungal diversity, acetylcholinesterase inhibitory activity, antioxidant activity, bioactivities

## Abstract

Wild *Polygala crotalarioides* is an ethnomedicinal plant traditionally used for neurological health. Endophytic fungi associated with medicinal plants are increasingly recognized as sources of bioactive natural products; however, their diversity and biological activities in wild *P. crotalarioides* remain poorly understood. We used high-throughput sequencing and culture-dependent methods to explored the diversity, community composition, and bioactivities of endophytic fungi associated with wild *P. crotalarioides*. Culturable isolates were identified via ITS sequencing and further screened for AChE-inhibitory and antioxidant activities. Our results revealed that a total of 818 fungal OTUs were detected, revealing pronounced tissue-specific community differentiation. Stem tissues harbored the highest fungal diversity, richness, and number of unique OTUs. Ascomycota and Basidiomycota dominated fungal communities across all tissues, while distinct biomarker taxa were enriched in different organs. Functional annotation indicated marked tissue-dependent variation in trophic composition. Among 108 culturable isolates, *Penicillium* sp. PRP024 and *Trichoderma* sp. PRP008 exhibited the strongest AChE inhibitory activities (>80%), whereas *Fusarium* sp. PSP053, *Diaporthe* sp. PSM027 and *Exserohilum* sp. PSP042 showed notable antioxidant activities. Wild *P. crotalarioides* hosts highly diverse and tissue-specific endophytic fungal communities. Several isolates exhibited promising acetylcholinesterase inhibitory and antioxidant activities, highlighting medicinal plant-associated endophytic fungi as potential sources of antioxidative natural products.

## 1. Introduction

Medicinal plants are indispensable reservoirs of bioactive substances and serve as core materials in global traditional medicine, providing abundant compounds for modern pharmaceutical and healthcare product development [1,2,3]. Endophytic fungi ubiquitously colonize the internal tissues of healthy host plants and form stable symbiotic relationships with their hosts [4,5]. Accumulating ethnopharmacological and phytochemical evidence confirms that endophytic fungi derived from medicinal herbs can synthesize secondary metabolites structurally and functionally similar to host plant components, possessing diverse pharmacological properties [6,7]. Accordingly, endophytic fungi have emerged as a promising and untapped microbial resource for exploring novel natural bioactive compounds from traditional medicinal plants.

The *Polygala* (Polygalaceae), consisting of approximately 500 species worldwide, with over 39 species distributed in China [8], is a well-recognized medicinal plant group in traditional folk medicine [8,9], with prominent effects on neuroprotection, antioxidation, memory improvement and anti-inflammation [10,11]. In the past three to five years, increasing studies have focused on the phytochemical profiles, pharmacological mechanisms and quality evaluation of *Polygala* species. Multiple bioactive constituents, including triterpene saponins, xanthones, oligosaccharide esters, flavonoids and coumarins, have been extensively identified from *Polygala* plants. Among them, tenuifolin, polygalaxanthone III and 3,6′-disinapoyl sucrose are widely adopted as standard quality control markers [8,9]. Modern pharmacological studies have further demonstrated that *Polygala* extracts and monomeric compounds exert remarkable neuroprotective effects against Alzheimer’s disease (AD) by inhibiting Aβ aggregation, suppressing tau hyperphosphorylation, relieving oxidative stress, and regulating cholinergic and neuroinflammatory pathways [12,13]. Given the critical roles of oxidative stress and cholinergic dysfunction in AD progression, acetylcholinesterase (AChE) has emerged as a key therapeutic target, and natural antioxidants are considered promising intervention candidates [14,15,16]. However, currently available AChE inhibitors, such as donepezil, rivastigmine, and galantamine, are frequently associated with gastrointestinal, cardiovascular, and neurological adverse effects, which may reduce patient adherence and restrict long-term therapeutic efficacy [16,17]. Consequently, screening for natural AChE inhibitors from plant endophytes has become a research priority for developing safe and novel neuroprotective agents. Given the inherent antioxidant and neuroprotective properties of *Polygala* plants, their symbiotic endophytic fungi are considered excellent candidates for mining bioactive metabolites with AChE-inhibitory and antioxidant activities. Nevertheless, existing studies on *P. crotalarioides* are merely confined to cultivation techniques and preliminary phytochemical analysis. Notably, the community composition, tissue distribution and temporal variation in endophytic fungi associated with wild *P. crotalarioides* across different plant tissues and growth stages remain unexplored.

In addition to pharmacological studies, recent research has also explored the genetic diversity, resource conservation, and sustainable utilization of *Polygala* medicinal materials [18,19]. *Polygala crotalarioides*, a perennial herb endemic to Southwest China, is locally used as a traditional medicinal plant [20]. Our research team has previously explored the artificial cultivation and basic biological characteristics of this species, providing a foundation for its sustainable resource utilization [21]. Nevertheless, existing studies on *P. crotalarioides* remain confined to cultivation techniques and preliminary phytochemical analysis; the community composition, tissue distribution, and temporal variation in endophytic fungi associated with wild *P. crotalarioides* across different tissues and growth stages remain entirely unexplored.

The integration of culture-independent high-throughput sequencing (HTS) and traditional culture-dependent isolation has been widely adopted as a complementary approach for comprehensively exploring the diversity and bioresource potential of endophytic fungi [22,23]. HTS enables comprehensive characterization of the diversity and community structure of plant endophytic fungi, whereas culture-dependent isolation provides living fungal strains for downstream bioactivity screening, metabolite discovery, and pharmacological validation [21]. To date, studies on wild *P. crotalarioides* have mainly focused on the characterization of endophytic fungal community diversity, whereas investigations integrating spatiotemporal community dynamics with the bioactivity evaluation of cultivable endophytic fungi remain limited.

Based on the above findings and our previous studies on cultivated *P. crotalarioides* [21], two key questions remain unresolved: (i) how endophytic fungal communities are distributed among different tissues of wild *P. crotalarioides* and whether tissue-specific differences exist; and (ii) whether cultivable endophytic fungi from wild *P. crotalarioides* possess antioxidant and AChE inhibitory activities. Addressing these questions is important for understanding the endophytic fungal resources associated with this ethnomedicinal plant and their potential functional significance.

Therefore, the present study integrated HTS and culture-dependent isolation to systematically explore the endophytic fungi inhabiting the leaves, stems, and roots of wild *P. crotalarioides*. Community richness, diversity, and dominant fungal taxa were characterized across different plant tissues, and the isolated fungal strains were further evaluated for DPPH and ABTS radical scavenging activities as well as AChE inhibitory activity in vitro. This study aimed to reveal the endophytic fungal community characteristics of wild *P. crotalarioides* and identify cultivable fungal resources with potential antioxidant and AChE inhibitory activities, thereby providing a basis for future studies on the functional exploitation of endophytic fungi associated with medicinal *Polygala* species.

## 2. Materials and Methods

### 2.1. Sample Collection

Wild *Polygala crotalarioides* plants were sampled in May 2022 from Shangzhai Natural Village, Cangyuan County, Yunnan, China (23°08′48.16″ N, 99°14′46.31″ E). Three independent 3 × 3 m plots (≥150 m apart) were established, and five healthy plants were randomly selected per plot. Root, stem, leaf, and flower tissues were collected separately and pooled by organ type within each plot to generate one composite sample per plot, yielding three biological replicates per tissue. Fresh samples were transported to the laboratory under sterile, low-temperature conditions and processed within 24 h. A portion of each sample was used directly for high-throughput sequencing, and the remainder was processed aseptically for endophytic fungal isolation. Sample codes followed the format: R (root), S (stem), L (leaf), and F (flower).

### 2.2. Sample Processing

Roots and aerial tissues were separated using sterile scissors, and surface soil was removed by rinsing with sterile water. Samples were surface-sterilized by sequential immersion in 75% ethanol (2 min), 2.5% sodium hypochlorite (NaClO) solution containing 0.1% Tween 80 (5 min), and 75% ethanol (30 s), followed by three rinses with sterile water and drying with sterile filter paper [22]. The effectiveness of sterilization was verified by spreading 100 μL of the final rinse solution on PDA medium and incubating at 28 °C for 24 h; absence of colony formation confirmed successful surface disinfection [21]. Sterilized tissues were cut into 0.5 cm fragments under aseptic conditions. Intact lesion-free samples (3 g) were weighed, transferred into sterile 50 mL tubes, and stored at −80 °C until DNA extraction.

### 2.3. High-Throughput Sequencing Analysis

#### 2.3.1. DNA Extraction and PCR Amplification

All samples’ total DNA was extracted from plant tissues using the E.Z.N.A.^®^ Plant DNA Kit (Omega Bio-Tek, Norcross, GA, USA) following the manufacturer’s protocol. Extraction blanks (sterile deionized water) were included to monitor cross-contamination. DNA integrity was assessed by 1% agarose gel electrophoresis, and DNA concentration and purity were measured using a NanoDrop 2000 spectrophotometer (Thermo Fisher Scientific, Waltham, MA, USA).

Fungal ITS fragments were amplified using primers ITS1F (5′-CTTGGTCATTTAGAGGAAGTAA-3′) and ITS2R (5′-GCTGCGTTCTTCATCGATGC-3′) [24] (Thermo Fisher Scientific, Waltham, MA, USA). Each 20 µL reaction contained 4 µL of 5× FastPfu buffer, 2 µL of 2.5 mM dNTPs, 0.8 µL of each primer (5 µM), 0.4 µL of FastPfu DNA polymerase (TransGen Biotech, Beijing, China), and 10 ng of template DNA. Thermal cycling consisted of initial denaturation at 95 °C for 3 min; 27 cycles of 95 °C for 30 s, 55 °C for 30 s, and 72 °C for 45 s; and a final extension at 72 °C for 10 min. Negative controls (sterile water replacing template DNA) were included in each batch. Amplicons were verified by 2% agarose gel electrophoresis, and paired-end sequencing was performed on the Illumina NextSeq 2000 platform at Majorbio Bio-Pharm Technology Co., Ltd. (Shanghai, China). Raw reads were deposited in the NCBI Sequence Read Archive (SRA) under BioProject accession PRJNA1207743, with sample accessions ranging from SRR31976288 to SRR31976299.

#### 2.3.2. Sequence Processing and OTU Clustering

Raw sequencing data were quality-filtered using fastp v0.20.0 [25]. with a sliding window (50 bp) quality threshold of Q20; reads shorter than 50 bp or containing ambiguous bases were discarded. High-quality paired-end reads were assembled using FLASH v1.2.7 [26] with default parameters (minimum overlap: 10 bp; maximum mismatch ratio: 0.2). Valid sequences were demultiplexed according to barcode sequences, allowing up to two primer mismatches. Clean reads were clustered into operational taxonomic units (OTUs) at a 97% similarity threshold using UPARSE v7.1 [27], with the threshold adopted following standard practice for fungal ITS metabarcoding. Non-fungal sequences (chloroplast and mitochondrial origin) were excluded. Taxonomic annotation was performed against the UNITE fungal ITS database (v8.0) [28] using the RDP Classifier (v2.14) with a 70% bootstrap confidence threshold [29]. To minimize biases from uneven sequencing depth, the OTU table was rarefied to the minimum valid sequence count per sample.

#### 2.3.3. Statistical Analysis

α- Diversity indices (Shannon, Simpson, Chao1, and ACE) were calculated using mothur v1.48.0 [30]. Normality was assessed using the Shapiro–Wilk test. For non-normally distributed data, group differences were evaluated using the Kruskal–Wallis test followed by Dunn’s post hoc test. β-Diversity was assessed via principal coordinate analysis (PCoA) based on Bray–Curtis distances. Community structure differences were statistically tested using ANOSIM and PERMANOVA (999 permutations). Linear discriminant analysis effect size (LEfSe) was employed to identify stage- and tissue-specific biomarkers (LDA score ≥ 2.5, *p* < 0.05). Fungal ecological guilds were assigned using FUNGuild v1.1 [31]. All bioinformatic and statistical analyses were performed in R v4.5.2 using the vegan v2.6-4, ggplot2 v3.4.4, VennDiagram v1.7.3, pheatmap, and UpSetR v1.4.0 packages [32]. Statistical significance was set at *p* < 0.05, with *p* < 0.01 considered highly significant.

### 2.4. Isolation and Characterization of Culturable Endophytic Fungi

Surface-sterilized tissues were cut into 3–5 mm fragments and placed on PDA medium (5–8 fragments per plate). Plates were incubated at 28 °C for at least 10 days; emerging fungal colonies were subcultured onto fresh PDA and preliminarily classified based on colony morphology [33]. Owing to limited flower tissue availability, the number of isolates obtained from these samples was restricted. Purified strains were preserved in 50% (*v*/*v*) glycerol at −80 °C.

Fungal genomic DNA was extracted using a modified CTAB method [34] with reference to the Omega fungal DNA extraction kit protocol. DNA quality and concentration were assessed using a NanoDrop spectrophotometer, retaining samples with DNA concentration ≥ 100 ng/25 μL. The ITS region was amplified with primers ITS1 and ITS4 [35]. The 60 μL PCR system contained 42.6 μL sterile ddH_2_O, 6 μL 10× buffer, 1.2 μL 2 mmol dNTPs, 1.5 μL of each 10 pmol primer, 1.2 μL 5 U Taq polymerase and 6 μL DNA template. Amplification conditions were set as initial pre-denaturation at 94 °C for 5 min; 35 cycles of 94 °C for 90 s, 53 °C for 90 s and 72 °C for 2 min; followed by final extension at 72 °C for 10 min. Amplicons were verified by 1% agarose gel electrophoresis and sent to Kunming Shuoqing Biotechnology Co., Ltd. (Kunming, China) for Sanger sequencing. Obtained sequences were compared against the GenBank database via BLAST 2.15. Isolates exhibiting sequence similarity below 95% were tentatively considered unidentified species; those with ≥95% similarity were assigned to genus level, and sequences with ≥97% identity were tentatively classified to species level. All sequences have been deposited in GenBank under accession numbers PV175970–PV176077.

### 2.5. Evaluation of Biological Activity

#### 2.5.1. Strain Fermentation and Extract Preparation

For metabolite production, solid-state fermentation was performed in glass culture vessels (5.7 cm internal diameter × 9.2 cm height), each containing 75 g of commercially available white rice and 75 mL of distilled water, with 1 mL of distilled water replaced by trace element solution to maintain a final liquid volume of 75 mL and a strict 1:1 (*w*/*v*) ratio of rice to liquid. The trace element solution contained, per liter: MgSO_4_·7H_2_O 0.01 g, CuSO_4_·5H_2_O 0.005 g, and FeSO_4_·7H_2_O 0.01 g. The vessels were sealed with breathable membrane closures to allow gas exchange while maintaining sterility. Following autoclaving at 121 °C for 20 min and cooling to room temperature, each vessel was inoculated with five agar plugs (5 mm diameter) taken from the actively growing margin of 7-day-old PDA cultures. Inoculated cultures were incubated at 28 °C in the dark for 28 days without agitation. After fermentation, 150 mL of methanol was added to each vessel, and the mixture was ultrasonicated for 1 h at room temperature; this extraction was repeated three times. The combined methanolic extracts from each sample were filtered through No. 1 filter paper and concentrated under reduced pressure using a rotary evaporator at 40 °C. The resulting crude extracts were dried at 50–55 °C to constant weight and stored at 4 °C until further analysis. Extraction yield was calculated as:

(dry weight of crude extract/initial rice medium weight) × 100%.

For conspecific endophytic fungal isolates recovered from wild *P. crotalarioides*, a single representative strain per species was selected for subsequent bioactivity assays. All crude fungal extracts were preliminarily screened at 2000 μg/mL to assess acetylcholinesterase (AChE) inhibitory activity and antioxidant potential. Isolates exhibiting strong dual bioactivities underwent further quantitative analysis to calculate their half-maximal inhibitory concentration (IC_50_) values. A total of 21 fungal strains were subjected to fermentation extraction and activity screening, with the corresponding strain information and extraction rates listed in Table S1.

#### 2.5.2. AChE Inhibitory Activity Assay

Acetylcholinesterase (AChE) inhibitory activity was measured using a modified Ellman colorimetric method [36]. Fermented rice extracts were dissolved in Dimethyl sulfoxide (DMSO, Sinopharm Chemical Reagent Co., Ltd., Shanghai, China) and serially diluted with water to concentrations of 40, 20, 15, 10, 5 and 2.5 mg/mL, then filtered through 0.45 μm membranes. The final DMSO proportion in reaction solution was controlled at 0.10%, with 2.0% DMSO contained in stock samples. Galantamine (Sigma-Aldrich, St. Louis, MO, USA) solution (1 mg/mL) served as positive control, 2.0% DMSO as negative control, and blank culture medium as blank control. The reaction mixture was prepared in 96-well plates (Corning Inc., Corning, NY, USA) by sequentially adding 110 μL pH 8.0 phosphate buffer (Sinopharm Chemical Reagent Co., Ltd., Shanghai, China), 10 μL sample solution and 40 μL 0.1 U/mL AChE solution (Shanghai Yuanye Bio-Technology Co., Ltd., Shanghai, China). After 20 min incubation at 37 °C, 20 μL 6.25 mmol/L 5,5’-Dithiobis(2-nitrobenzoic acid) (DTNB, Sigma-Aldrich, St. Louis, MO, USA) and 20 μL 6.25 mmol/L Acetylthiocholine iodide (ATChI, Sigma-Aldrich, St. Louis, MO, USA) were supplemented. Absorbance at 405 nm was detected within one hour using an ELISA reader (Tecan Trading AG, Männedorf, Switzerland). All assays were performed in triplicate. AChE inhibition rate was calculated as:

Inhibition rate (%) = (A_NC_ − A_S_)/A_NC_ × 100%.

where A_NC_ and A_S_ represent absorbance of the negative control and test sample, respectively. The IC50 was computed with GraphPad Prism v8.0.2 (GraphPad Software, San Diego, CA, USA), and raw data were sorted in Microsoft Excel.

#### 2.5.3. DPPH Radical Scavenging Activity Assay

(2,2′-diphenyl-1-picrylhydrazyl) (DPPH) radical scavenging activity was determined using a modified DPPH assay according to Akinsanya, M.A. et al. [37]. DPPH powder (Sigma-Aldrich, St. Louis, MO, USA) was dissolved in methanol to prepare a 0.2 mM working solution. Test samples and the positive control (ascorbic acid, vitamin C; Sigma-Aldrich, St. Louis, MO, USA) were serially diluted with methanol to concentrations of 0.25–4 mg/mL and 0.1–1.0 mg/mL, respectively. A 100 μL aliquot of each sample solution was mixed with 100 μL of DPPH working solution in 96-well microplates. After incubation in the dark at room temperature for 30 min, the absorbance of the reaction mixture was measured at 515 nm using a microplate reader. The DPPH radical scavenging rate was calculated using the following formula:

Scavenging rate (%) = (A_C_ − A_S_)/A_C_ × 100%.

where A_C_ is the absorbance of the blank control (methanol and DPPH solution without test samples or positive controls), and A_S_ is the absorbance of reaction systems supplemented with samples or vitamin C standards. Raw data were analyzed using Microsoft Excel 2021, graphs were plotted with Origin 2024, and IC_50_ values were calculated via GraphPad Prism v8.0.2.

#### 2.5.4. ABTS Radical Scavenging Activity Assay

The 2,2’-azinobis(3-ethylbenzothiazoline-6-sulfonicacid) (ABTS) radical scavenging activity was measured in this assay [38,39]. ABTS (Sigma-Aldrich, St. Louis, MO, USA) radical cation was generated by mixing ABTS stock solution with 2.45 μM potassium persulfate (Sinopharm Chemical Reagent Co., Ltd., Shanghai, China), and the mixture was incubated in darkness at room temperature for 12–16 h before use. The prepared solution was diluted with methanol to obtain an absorbance value of 0.700 ± 0.02 at 734 nm, followed by equilibration at 30 °C. Fresh solution was newly prepared for each independent test. Rice fermentation extracts were dissolved in methanol and serially diluted to concentrations of 40, 20, 15, 10, 5 and 2.5 mg/mL. Vitamin C was adopted as a positive control with concentrations ranging from 0.1 to 1.0 mg/mL. For the reaction setup, 10 μL of the sample solution was blended with 190 μL of the solution. After 7 min of incubation, absorbance was measured at 734 nm. The radical scavenging rate was calculated as follows:

Scavenging rate (%) = (A_C_ − A_S_)/A_C_ × 100%.

where A_C_ refers to the absorbance of the blank group containing ABTS radical and methanol, and A_S_ represents the absorbance of the sample or control reaction system. Raw data were analyzed via Microsoft Excel 2021, graphs were plotted using Origin 2024, and IC50 values were calculated with GraphPad Prism 8.0.2.

## 3. Results

### 3.1. Fungal ITS Gene Sequencing Data

After filtering low-quality and duplicate reads, 538,528 high-quality sequences were retained, with an average length of 243 bp. All samples were normalized to the minimum read count of 31,294 for downstream analysis, and detailed sequencing information is presented in Table S2. Sequences were clustered into operational taxonomic units (OTUs) at a 97% similarity threshold. After removing sequences affiliated with chloroplasts and mitochondria, 818 non-redundant OTUs were obtained in total (Table S3). The rarefaction curves of samples from different plant organs gradually plateaued with increasing sequencing depth (Figure S1). The Good’s coverage of all samples exceeded 99%, indicating that the sequencing adequately captured the endophytic fungal communities of wild *Polygala crotalarioides*.

### 3.2. α-Diversity of Fungal Communities Across Tissues

**Figure 1:**
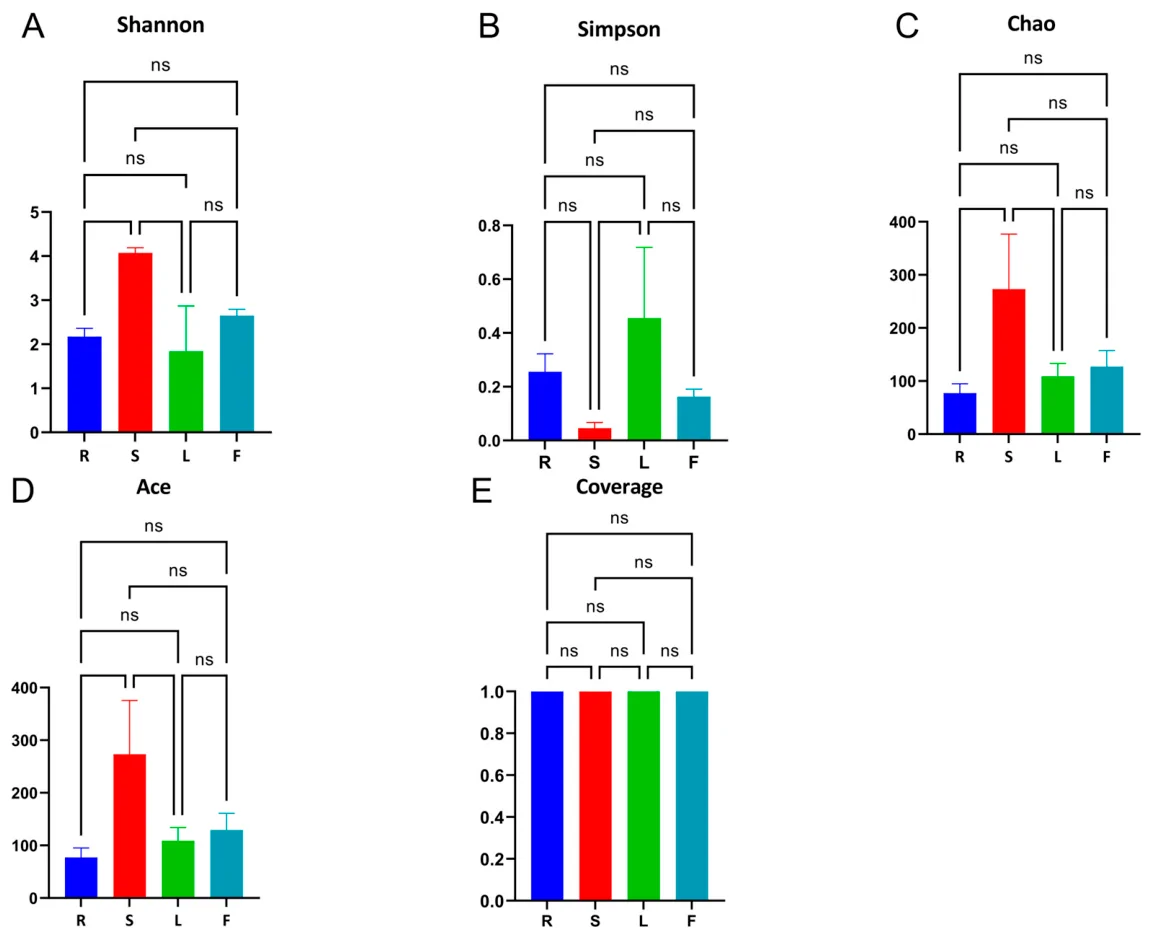
Fungal diversity and richness indices of endophytic communities in different tissues of wild *Polygala crotalarioides*. (**A**) Shannon diversity index, (**B**) Simpson diversity index, (**C**) Chao richness index, (**D**) Ace richness index, and (**E**) Good’s coverage index. Bars represent the mean ± standard deviation (SD) of three biological replicates. Different asterisks indicate significant differences between groups (Kruskal–Wallis test, ns, not significant, *p* > 0.05). R, root; S, stem; L, leaf; F, flower.

We compared the alpha diversity of endophytic fungi in different tissues of wild *P. crotalarioides* (Figure 1). Alpha diversity differed significantly among tissues (*p* < 0.05). Stems had the highest Shannon index and the lowest Simpson index, indicating markedly higher fungal diversity than roots, leaves and flowers. Meanwhile, stems exhibited the maximum ACE and Chao values for community richness, with significant differences observed relative to the other three tissues (*p* < 0.05). Collectively, stems possessed the greatest fungal diversity and richness, followed by flowers with moderate diversity, roots with intermediate indices, and leaves with the lowest diversity and richness.

### 3.3. Composition of Endophytic Fungal Communities in Wild Polygala crotalarioides

**Figure 2:**
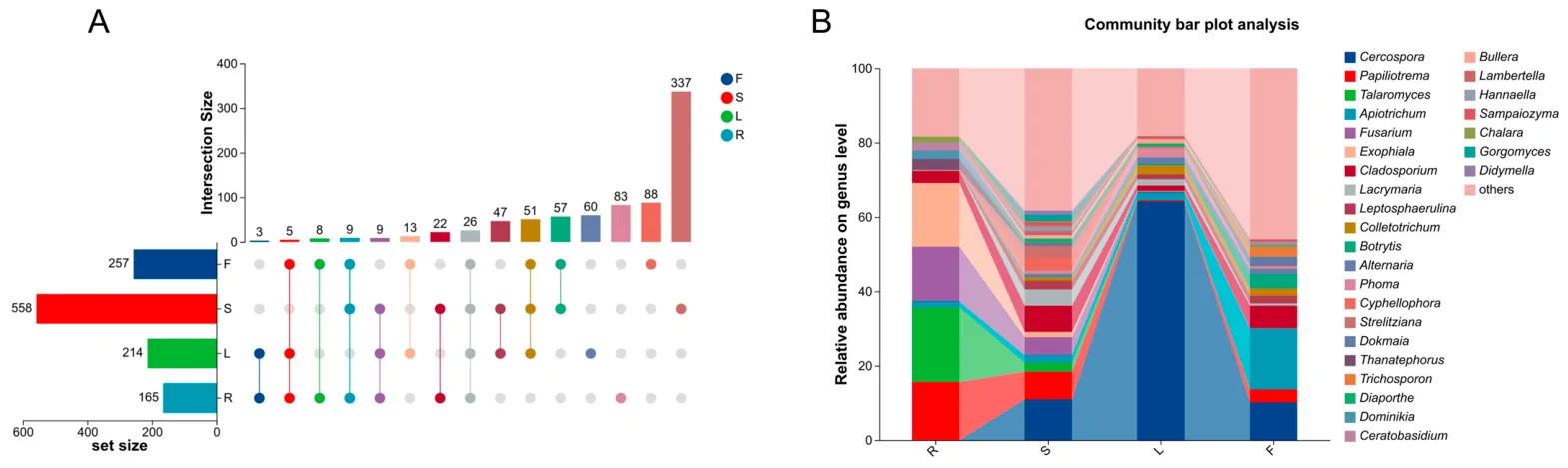
Endophytic fungal community composition and shared taxa in different tissues of wild *Polygala crotalarioides*. (**A**) UpSet plot showing the number of shared and unique fungal OTUs among the root (R), stem (S), leaf (L), and flower (F) tissues. The horizontal bars represent the total number of OTUs in each tissue, and the vertical bars represent the size of the intersection. colored dots represent individual tissue groups: root (R, cyan), stem (S, red), leaf (L, green), flower (F, dark-blue). Colored connecting-lines link selected dots to indicate combinations of tissue groups, and each vertical bar above denotes the number of shared fungal OTUs within that intersection set. (**B**) Stacked bar plot showing the relative abundance of fungal genera in each tissue. The “others” category includes genera with relative abundance < 1% in all samples.

OTU profiling revealed pronounced tissue-specific structuring of endophytic fungal communities in wild *P. crotalarioides*. Stems harbored the highest overall fungal diversity and richness, and also exhibited the largest number of tissue-specific OTUs. Only a minimal proportion of OTUs was shared among all organs, indicating a highly restricted core mycobiome. These results suggest low inter-organ microbial overlap (Figure 2A).

Taxonomic annotation of representative OTU sequences assigned 818 OTUs to 9 phyla, 31 classes, 80 orders, 180 families, and 288 genera (Table S3). At the phylum level, wild *P. crotalarioides* endophytic fungi were mainly affiliated with Ascomycota and Basidiomycota across all tissues, respectively. The relative abundance of Ascomycota varied markedly among tissues: 65.15% in roots, 71.76% in stems, 85.04% in leaves and 44.22% in flowers. For Basidiomycota, the corresponding values were 26.36%, 24.59%, 8.07% and 26.47%. Minor phyla and unidentified fungal groups were detected in all tissues, and the relative abundance of other phyla was below the detectable threshold (Figures S2 and S3).

Genus-level analysis showed that the composition of dominant endophytic fungal genera varied distinctly across roots, stems, leaves and flowers. *Cercospora* was the absolute dominant genus in leaves. Roots were mainly dominated by *Talaromyces*, *Exophiala*, *Papiliotrema* and *Fusarium*. Stems harboured high abundances of *Cercospora* and *Papiliotrema*, whereas flowers were predominated by *Apiotrichum* and *Cercospora*. Most other genera had extremely low relative abundances in all tissues (Figure 2B).

**Figure 3:**
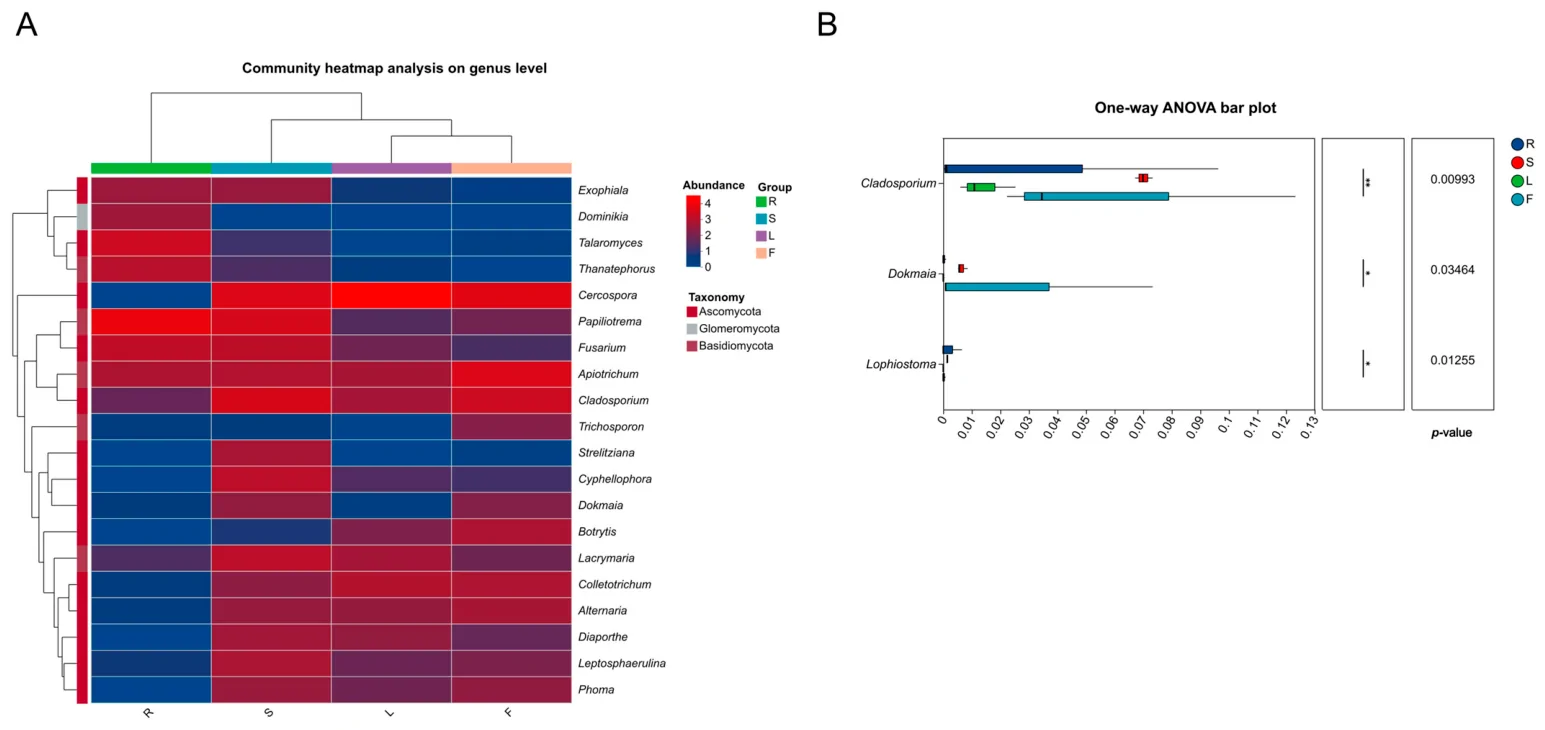
Genus-level abundance and differential analysis of endophytic fungi in wild *Polygala crotalarioides*. (**A**) Heatmap showing relative abundance of fungal genera across root (R), stem (S), leaf (L), and flower (F) tissues; color gradient indicates abundance from low (blue) to high (red). (**B**) One-way ANOVA bar plot displaying genera with significant abundance differences among tissues, with mean proportion (%) on the horizontal axis, standard deviation error bars, and significance levels (* *p* < 0.05; ** *p* < 0.01) indicated.

Horizontal cluster analysis at the genus level showed that endophytic fungal communities from leaves, stems, and flowers clustered together, indicating high compositional similarity among these three organs. By contrast, root communities formed a separate clade (Figure 3A). One-way ANOVA detected significant differences in the relative abundances of dominant genera across the four organs (*p* < 0.05). Specifically, *Cladosporium*, *Dokmaia* and *Lophiostoma* differed significantly among organs (*p* < 0.05). The relative abundance of *Cladosporium* presented a clear gradient across organs, while *Dokmaia* and *Lophiostoma* showed uneven distribution among different plant organs (Figure 3B).

### 3.4. β-Diversity of Endophytic Fungal Communities

**Figure 4:**
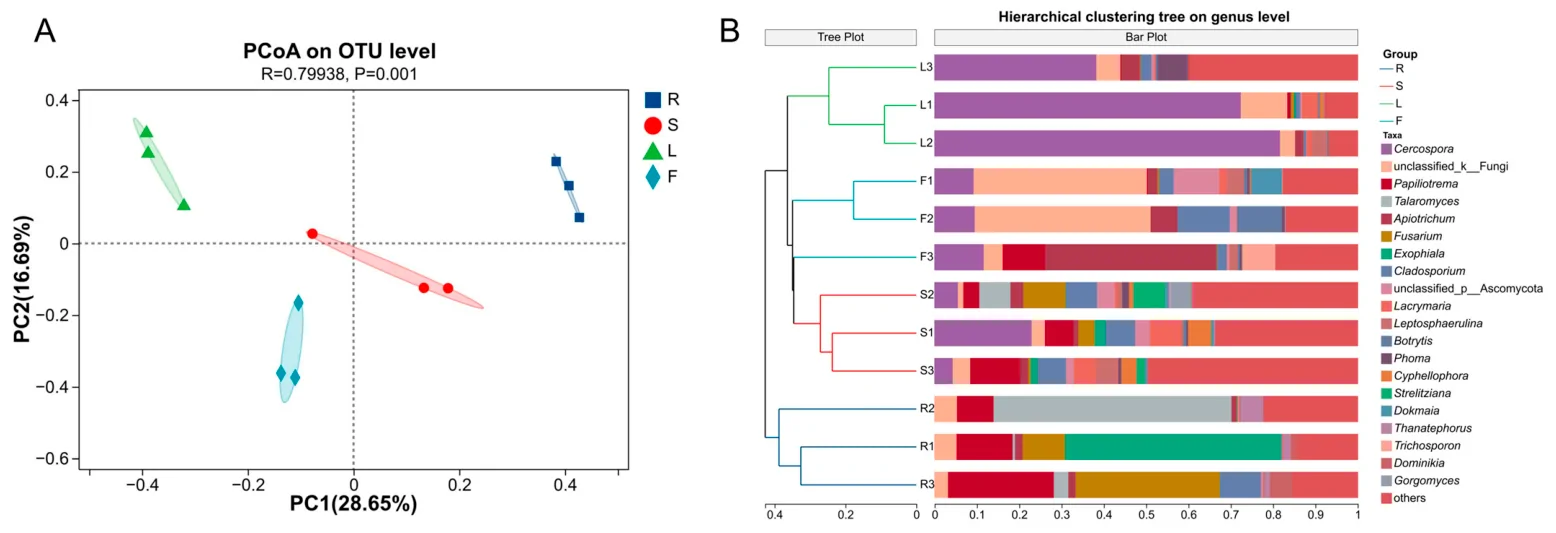
(**A**) Community dissimilarity analysis of endophytic fungal communities from four tissue compartments of wild *Polygala crotalarioides*. Axes represent the first (PC1, 28.05%) and second (PC2, 16.90%) principal coordinates, with values in parentheses indicating the percentage of explained variation. different colors and symbols represent four tissue groups: dark-blue squares for root (R), red circles for stem (S), green triangles for leaf (L), and cyan diamonds for flower (F). Proximity between points indicates similarity in community composition. ANOSIM results: R = 0.79938, *p* = 0.001. (**B**) Genus-level hierarchical clustering tree combined with stacked bar charts showing genus relative abundance of each replicate.

PCoA and hierarchical clustering analyses consistently demonstrated striking tissue-specific separation of endophytic fungal communities in wild *P. crotalarioides*. OTU-based PCoA ordination exhibited complete spatial segregation of root, stem, leaf and flower samples, and ANOSIM verified significant community dissimilarity across compartments (R = 0.78704, *p* = 0.001) (Figure 4A). Hierarchical clustering on genus-level profiles fully partitioned replicates by tissue type, with each cluster displaying unique dominant fungal genera (Figure 4B).

### 3.5. Analysis of Differentially Enriched Taxa of Fungal Communities Across Different Tissues

**Figure 5:**
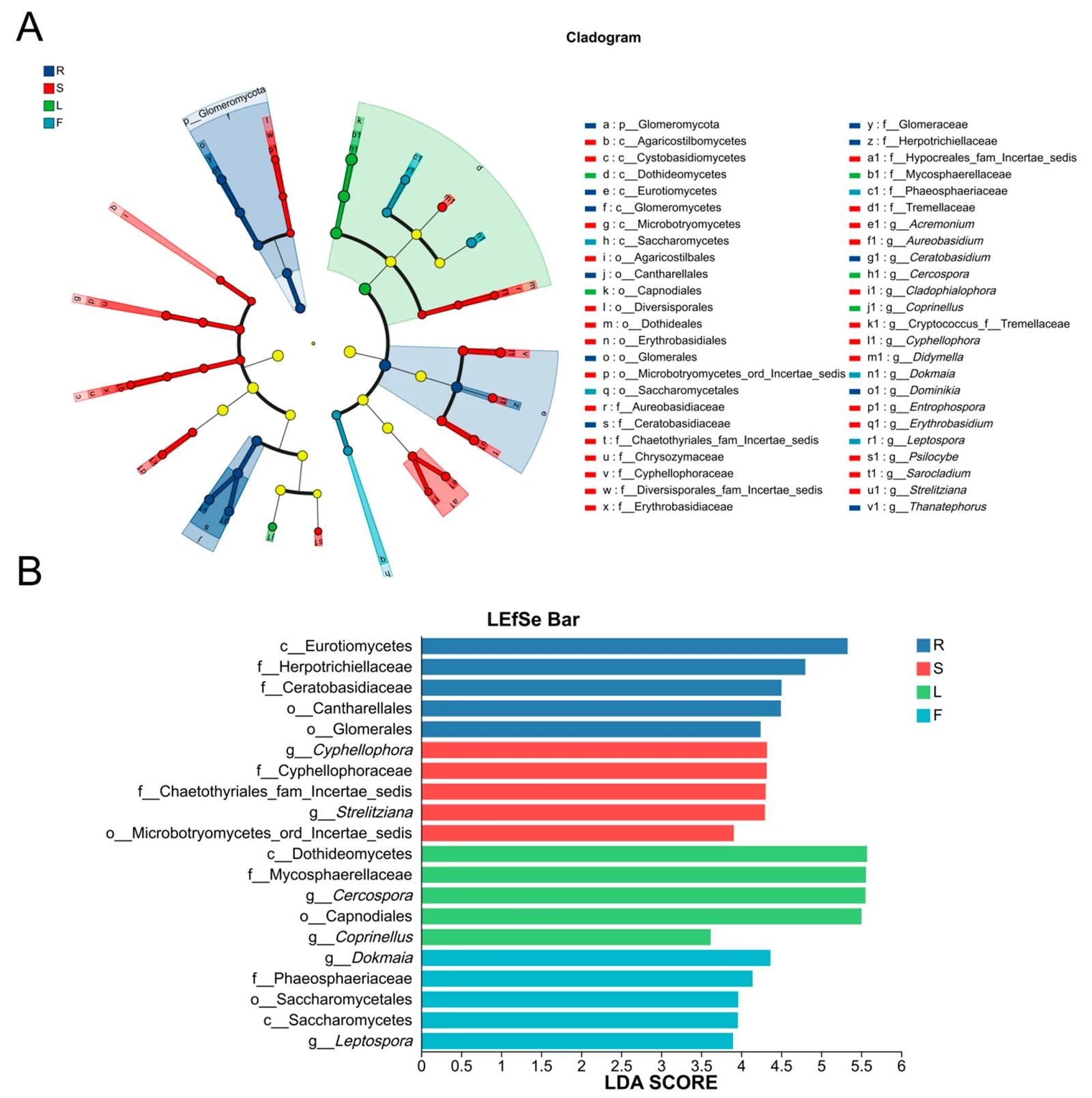
LEfSe analysis of tissue-specific biomarker fungi in root (R), stem (S) and leaf (L) compartments of wild *Polygala crotalarioides*. (**A**) Phylogenetic cladogram of differential fungal taxa; colors denote the enriched tissue for each taxonomic clade. (**B**) LDA score bar plot showing the discriminative taxa with LDA threshold > 2.5.

LEfSe analysis was conducted to screen tissue-specific biomarker fungi across four tissues (root, stem, leaf and flower) of wild *P. crotalarioides*. The results confirmed significant tissue-specific differentiation of endophytic fungal taxa, with distinct differential fungal populations enriched in roots, stems, and leaves, while no significantly differential fungal taxa were detected in flowers (Figure 5A). At multiple taxonomic levels, fungal communities exhibited differential enrichment patterns among various tissues. Root tissues harbored fungal biomarkers with high LDA scores, mainly including the class Eurotiomycetes, the order Cantharellales, the family Ceratobasidiaceae, and the genus *Thanatephorus*. At the class level, Eurotiomycetes and Cystobasidiomycetes were specifically abundant in root tissues. In stem tissues, multiple fungal taxa were significantly enriched, covering the orders Diaporthales and Hypocreales, the families Cyphellophoraceae, Trichomeriaceae, Didymosphaeriaceae and Diaporthaceae, as well as the genera *Cyphellophora*, *Articulospora* and *Diaporthe*. Leaf tissues possessed a unique assemblage of differential fungal biomarkers. The phylum Ascomycota, the class Dothideomycetes, the order Capnodiales, the family Mycosphaerellaceae, and the genera *Cercospora* and *Coprinellus* presented relatively high LDA scores and dominated the fungal community of leaves. All these tissue-enriched fungal taxa formed independent phylogenetic clades corresponding to specific plant tissues. Overall, the composition of differentially enriched endophytic fungi varied markedly among root, stem, and leaf tissues of wild *P. crotalarioides*, strongly demonstrating the distinct tissue-specific distribution characteristics of endophytic fungal communities in this plant (Figure 5B).

### 3.6. FUNGuild-Based Trophic Composition of Endophytic Fungi in Wild Polygala crotalarioides

**Figure 6:**
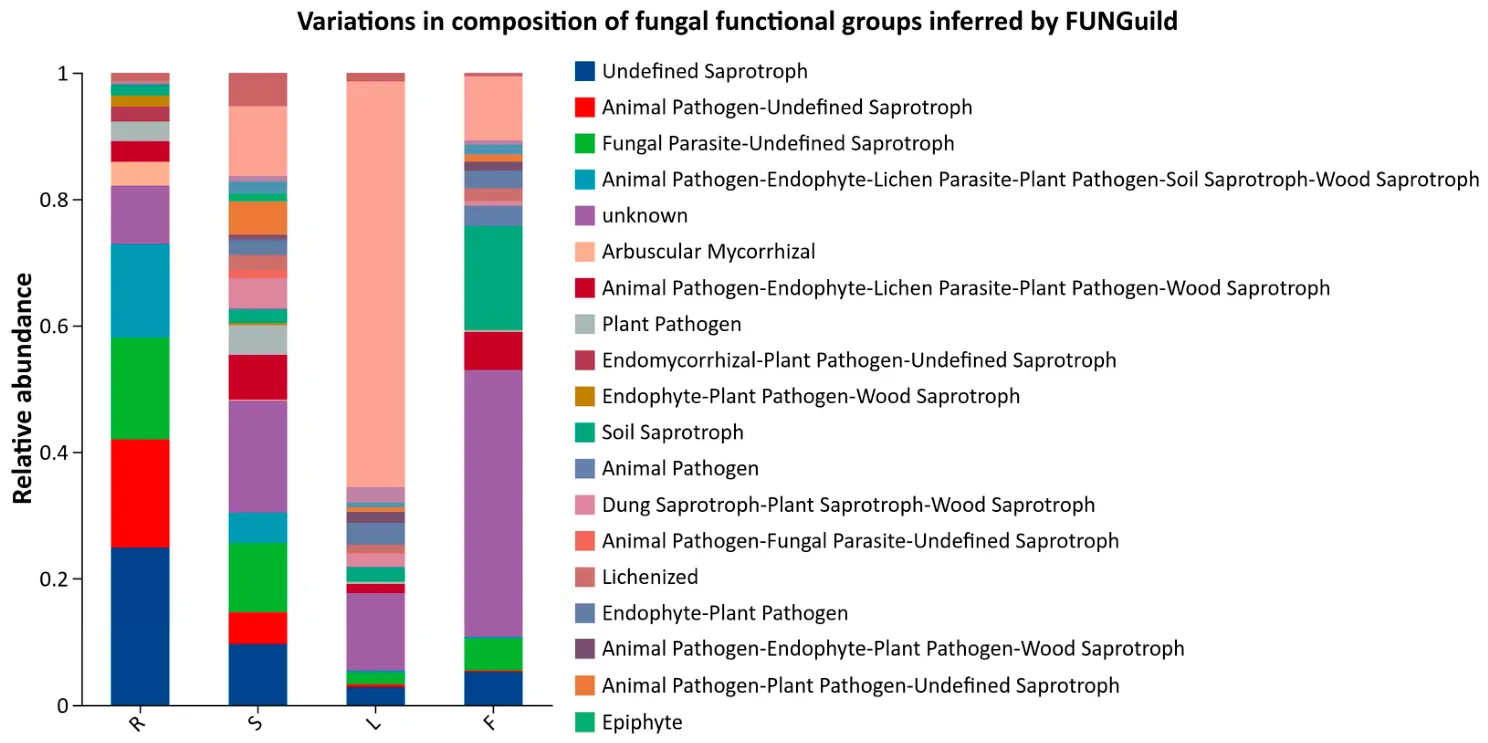
Fungal functional guild composition predicted via FUNGuild in four tissue compartments (R: root, S: stem, L: leaf, F: flower) of wild *Polygala crotalarioides*. Stacked bars show the relative abundance of each trophic functional group.

FUNGuild functional annotation demonstrated marked organ-dependent divergence in the trophic composition of endophytic fungi isolated from wild *P. crotalarioides*. Arbuscular mycorrhizal fungi dominated and peaked in relative abundance within leaf tissues, while undefined saprotrophs and animal-pathogenic fungi constituted the predominant functional fraction in roots. Floral fungal assemblages were chiefly composed of functionally unclassified fungi and soil saprotrophs, and stems accommodated the richest spectrum of trophic groups with relatively uniform abundance distribution across diverse functional guilds. A substantial fraction of unknown and undefined saprotrophic fungi was recovered in all four tested plant organs (Figure 6).

### 3.7. Isolation and Identification of Culturable Endophytic Fungi in Wild Polygala crotalarioides

In total, 108 endophytic fungal strains were isolated from the leaves, roots, and stems of wild *P. crotalarioides*, including 32 strains from leaves, 28 strains from roots, and 48 strains from stems. Based on ITS sequence identification, all isolates were taxonomically classified into 2 phyla, 4 classes, 9 orders, 14 families, and 17 genera. Among the identified fungi, *Colletotrichum* sp. and *Fusarium* sp. were isolated from all three tested organs with relatively high isolation frequencies (Table S4). The remaining fungal genera were recovered from only one or two plant organs.

**Figure 7:**
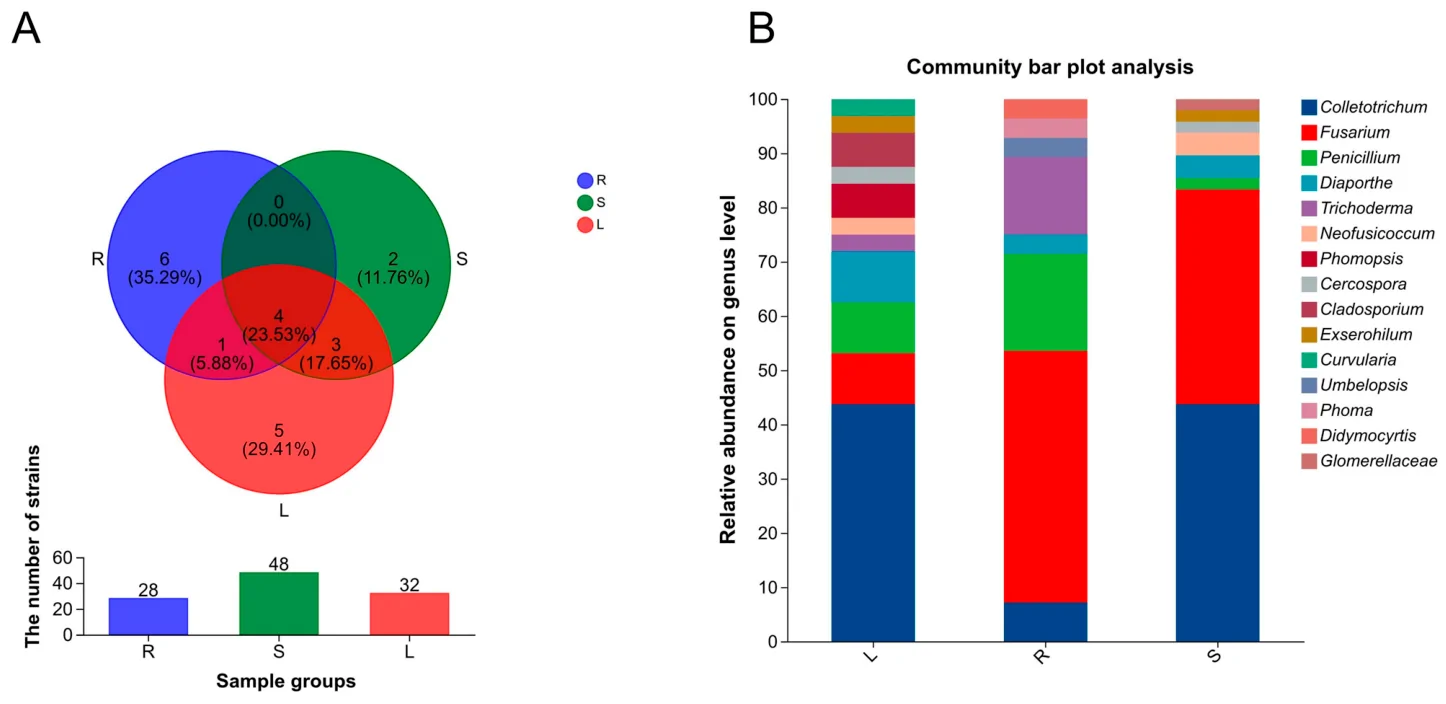
Venn diagram analysis and genus-level distribution of culturable endophytic fungi isolated from different tissues of wild *Polygala crotalarioides*. (**A**) Venn diagram illustrating the counts and proportions of unique and shared fungal genera across root (R), stem (S), and leaf (L) tissues. Numbers within each segment represent the number of genera, and percentages denote their proportions relative to the total number of detected genera. (**B**) Bar plot showing the genus-level relative abundance of fungal isolates pooled from all tissues (leaf, root and stem); each bar represents the percentage of isolates assigned to the corresponding genus.

Genus-level comparative analysis and Venn diagram analysis identified unique and shared fungal genera among leaf, root and stem tissues of wild *P. crotalarioides*. A total of 5, 6 and 2 tissue-specific genera were detected in leaves, roots and stems, respectively. Four genera, *Colletotrichum*, *Diaporthe*, *Fusarium* and *Penicillium*, were common to all three tissues. *Trichoderma* was shared exclusively by leaves and roots, while three genera including *Cercospora*, *Exserohilum*, and *Neofusicoccum* were shared between leaves and stems (Figure 7A and Table S5).

Fungal community composition exhibited slight differences across different plant tissues at the phylum level. Ascomycota and Mucoromycota were detected in root tissues, whereas only Ascomycota was present in leaf and stem tissues. Ascomycota dominated the fungal communities of roots, stems, and leaves, with relative abundances of 100%, 96.30% and 100%, respectively (Table S5).

Genus-level analysis revealed obvious differences in dominant fungal genera across leaf, root and stem tissues. Leaf tissues harbored 11 fungal genera, while both root and stem tissues contained 8 genera each. The dominant taxa varied greatly among organs: *Colletotrichum* showed the highest relative abundance in leaves; *Fusarium* predominated in roots; and *Colletotrichum* was the most abundant genus in stems (Figure 7B).

### 3.8. Bioactivity Screening of Endophytic Fungi

#### 3.8.1. Screening of AChE Inhibitory Activity and IC_50_ Determination

**Figure 8:**
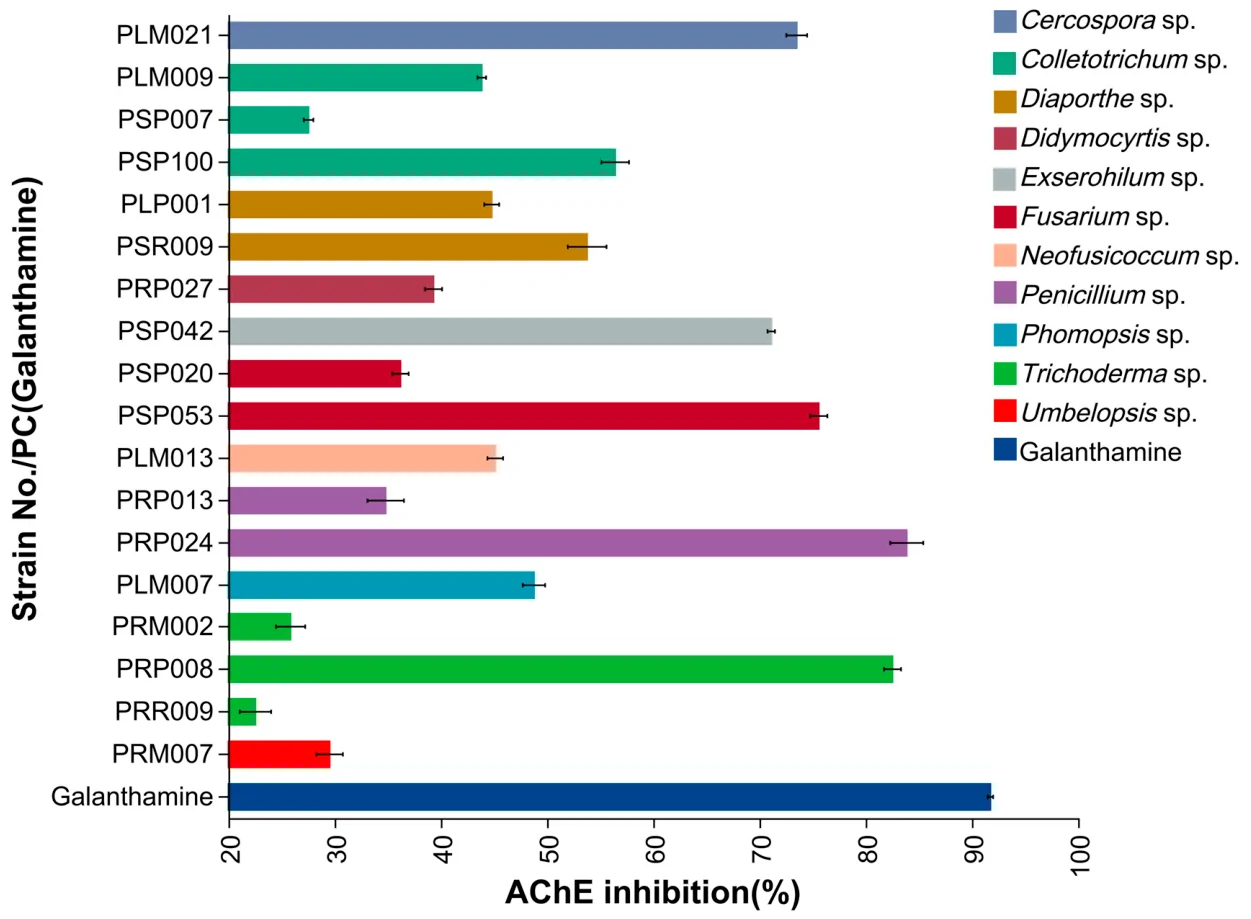
AChE inhibitory activity of endophytic fungal extracts from wild *Polygala crotalarioides*. The horizontal bars represent the mean percentage of AChE inhibition ± standard deviation (SD, n = 3) at a fixed concentration of 2000 μg/mL. Galanthamine (PC) was used as the positive control. The strain names corresponding to each code are listed in the legend.

Acetylcholinesterase (AChE) inhibitory activity was screened among endophytic fungi isolated from wild *P. crotalarioides*. Two strains exhibited the highest AChE inhibitory activity, with inhibition rates exceeding 80%: *Penicillium* sp. PRP024 (83.87 ± 1.51%) and *Trichoderma* sp. PRP008 (82.53 ± 0.80%). Five strains showed moderate-to-high activity (>50%), and the remaining 11 strains displayed low to moderate activity (22.55–49.0%). All assays showed high reproducibility, with standard deviations <2%. The IC_50_ values of seven high-activity strains for AChE inhibition were further determined. The lowest IC_50_ was recorded for *Cercospora* sp. PLM021 (418.1 μg/mL, 95% CI: 393.6–449.6 μg/mL), followed by *Trichoderma* sp. PRP008 (441.3 μg/mL, 95% CI: 441.9–470.2 μg/mL). IC_50_ values for the other strains ranged from 536.7 μg/mL (*Penicillium* sp. PRP024) to 827.5 μg/mL (*Diaporthe* sp. PSR009). The positive control galantamine had an IC_50_ of 67.5 μg/mL (95% CI: 64.00–71.08 μg/mL). All samples produced narrow 95% confidence intervals (Figure 8 and Table S6).

#### 3.8.2. DPPH Radical Scavenging Activity and IC_50_ Determination of Endophytic Fungi

**Figure 9:**
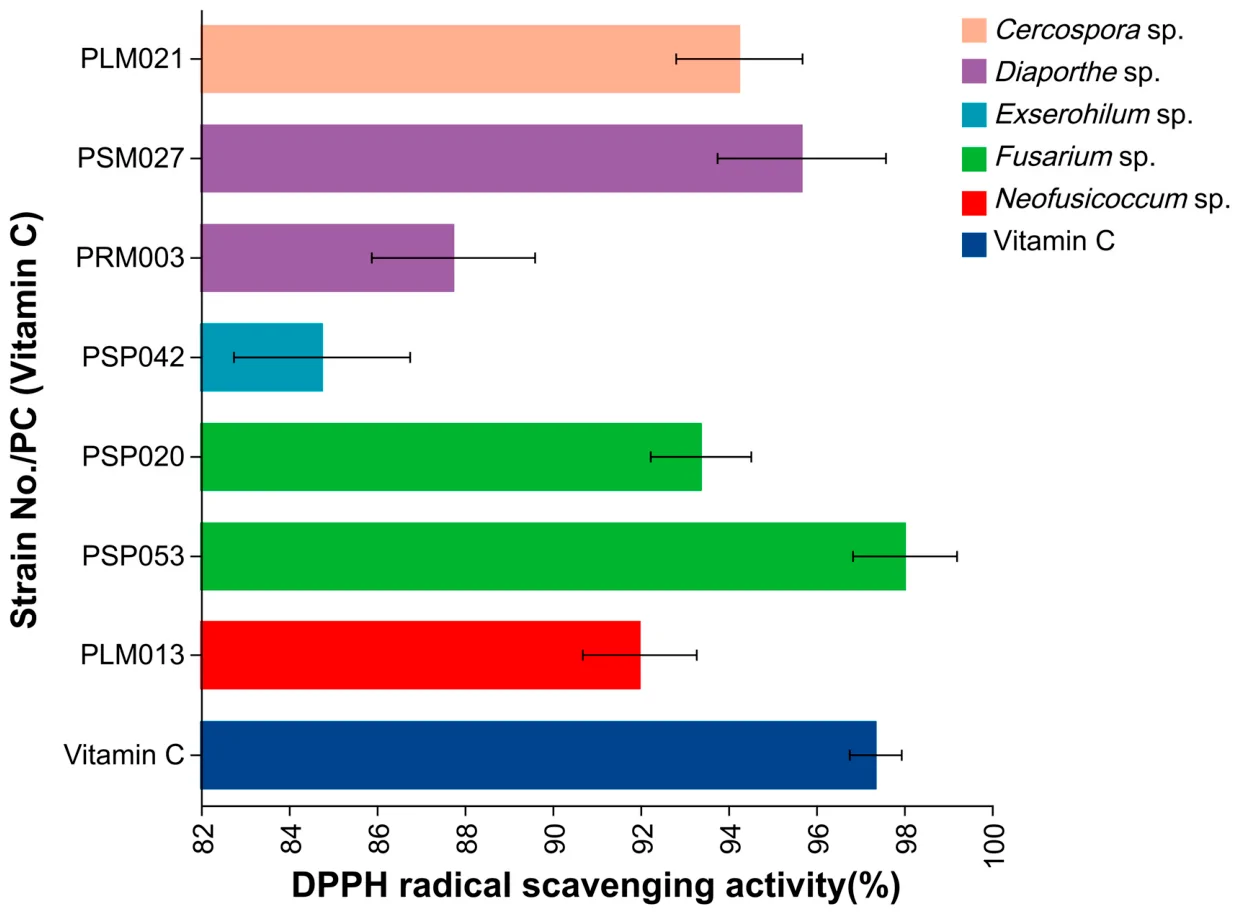
DPPH radical scavenging activity of endophytic fungal extracts from wild *Polygala crotalarioides*. The horizontal bars represent the mean percentage of DPPH radical scavenging activity ± standard deviation (SD, n = 3) at a concentration of 2000 μg/mL. Vitamin C (PC) was used as the positive control. The strain names corresponding to each code are listed in the legend.

(2,2-diphenyl-1-picrylhydrazyl) (DPPH) radical scavenging activity was also evaluated for these fungal strains. Seven strains showed high activity with scavenging rates ≥ 80%, and *Fusarium* sp. PSP053 (98.03 ± 1.19%) presented the strongest activity, followed by *Diaporthe* sp. PSM027 (95.67 ± 1.92%) and *Cercospora* sp. PLM021 (94.25 ± 1.44%). Standard deviations of all measurements were below 2%. The IC_50_ values for DPPH scavenging were measured in seven high-activity strains. *Exserohilum* sp. PSP042 had the lowest IC_50_ at 258.7 μg/mL (95% CI: 238.8–275.0 μg/mL), followed by *Fusarium* sp. PSP053 (436.0 μg/mL, 95% CI: 388.4–487.7 μg/mL) and *Fusarium* sp. PSP020 (466.6 μg/mL, 95% CI: 414.1–514.7 μg/mL). The IC_50_ values of the rest of the strains ranged from 693.6 μg/mL to 771.5 μg/mL. The positive control vitamin C showed an IC_50_ of 10.10 μg/mL (95% CI: 8.20–12.36 μg/mL), and all assays had narrow 95% confidence intervals (Figure 9 and Table S7).

#### 3.8.3. ABTS Radical Scavenging Activity and IC_50_ Determination of Endophytic Fungi

**Figure 10:**
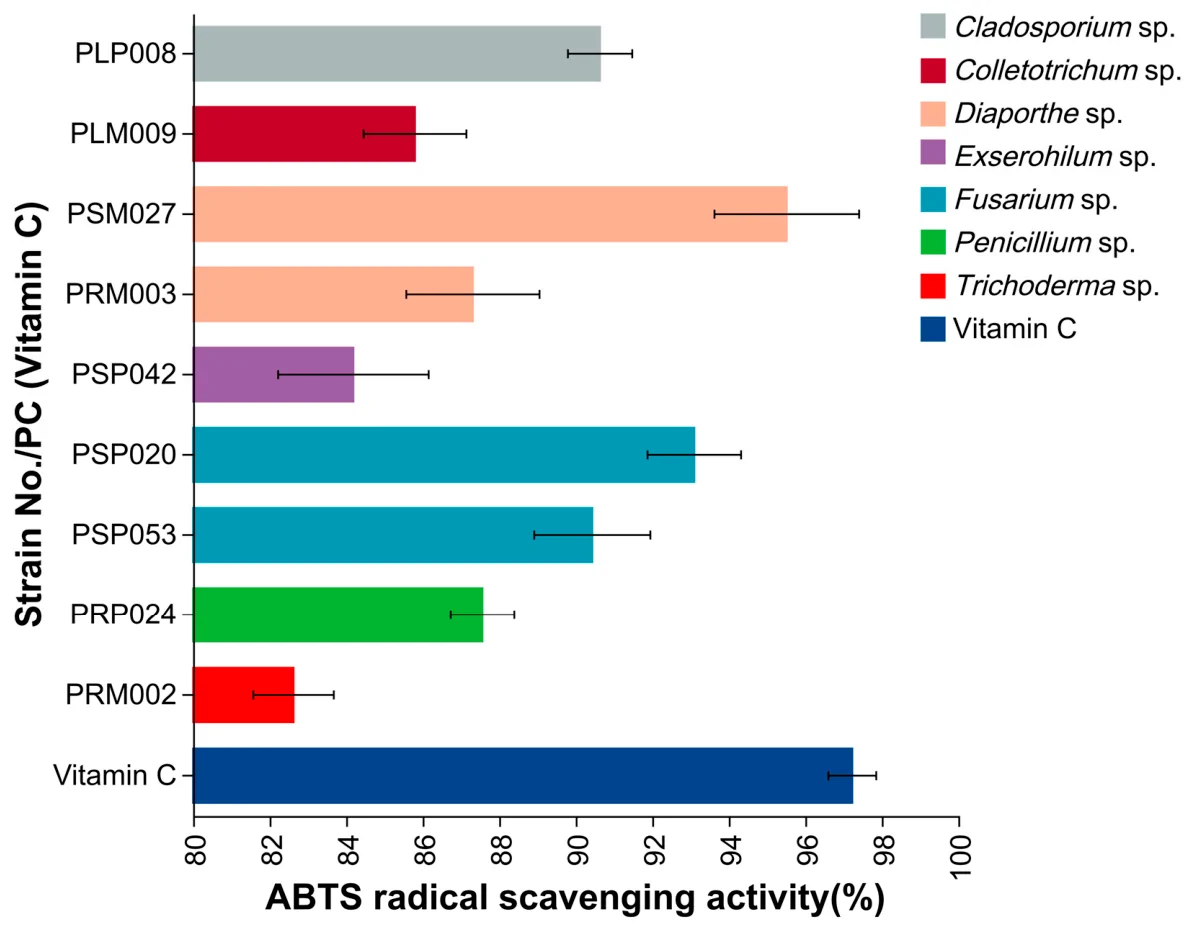
ABTS radical scavenging activity of endophytic fungal extracts from wild *Polygala crotalarioides*. The horizontal bars represent the mean percentage of ABTS radical scavenging activity ± standard deviation (SD, n = 3) at a fixed concentration of 2000 μg/mL. Vitamin C (PC) was used as the positive control. The strain names corresponding to each code are listed in the legend.

For the 2,2′-azinobis(3-ethylbenzothiazoline-6-sulfonicacid) (ABTS) radical scavenging activity, nine strains displayed high activity with scavenging rates ≥ 80%, among which *Diaporthe* sp. PSM027 (95.52 ± 1.89%) had the highest activity. All standard deviations were less than 2%. The IC_50_ values for ABTS scavenging were determined in nine high-activity strains. *Fusarium* sp. PSP053 yielded the lowest IC_50_ of 418.7 μg/mL (95% CI: 402.4–434.7 μg/mL), followed by *Penicillium* sp. PRP024 (423.2 μg/mL, 95% CI: 402.6–443.6 μg/mL). IC_50_ values of the remaining strains ranged from 585.7 μg/mL to 712.5 μg/mL. The positive control vitamin C had an IC_50_ of 70.06 μg/mL (95% CI: 42.16–83.80 μg/mL). Narrow 95% confidence intervals were observed across all tests, indicating good reproducibility (Figure 10 and Table S8).

## 4. Discussion

This study integrated high-throughput sequencing and culture-dependent approaches to characterize the endophytic fungal communities in different tissues of wild *Polygala crotalarioides* and to evaluate the antioxidant and AChE-inhibitory activities of cultivable isolates. The recovered strains were predominantly assigned to *Penicillium*, *Fusarium*, *Diaporthe*, and *Cercospora*, with several isolates displaying both antioxidant and AChE-inhibitory properties. Notably, wild-host-derived endophytes exhibited distinct community composition and bioactive profiles compared to those previously reported from cultivated *P. crotalarioides*.

α-diversity analysis revealed that stems of *P. crotalarioides* harbored the highest endophytic fungal richness and diversity among the four tissues examined (root, stem, leaf, and flower), as evidenced by the maximum Shannon, ACE, and Chao1 indices, the lowest Simpson index, and the highest total (558) and unique (337) OTU counts. This tissue-specific distribution is consistent with the widely reported niche differentiation of plant endophytes [40]. Several ecological factors may hypothetically contribute to the observed stem-biased fungal enrichment in wild *P. crotalarioides*. The stem tissues provide nutrient-transport pathways and heterogeneous carbon niches [41] that may favor fungal colonization. In contrast, leaf tissues are subject to senescence, high ultraviolet radiation, and strong environmental filtering [42,43], which may constrain fungal establishment. The low fungal diversity in flowers may be associated with the accumulation of defensive phytometabolites during anthesis, whereas the relatively lower complexity of root fungal communities could be limited by rhizosphere microbial competition and variable soil conditions. It should be noted, however, that the ecological interpretations proposed above remain hypothetical and require further experimental validation in future work.

Ascomycota predominated across all examined organs, matching widespread distribution patterns of endophytic fungi in terrestrial medicinal plants [44,45]. Variable Ascomycota relative abundances across tissues (85.04% leaf, 71.76% stem, 65.15% root, 44.22% flower) may reflect differential host selective pressure across discrete physiological microhabitats [46], while Basidiomycota mainly accumulated in root and flower fractions, predominantly comprising animal-pathogenic and soil saprotrophs taxa capable of intermittent microbial exchange with surrounding bulk soil [47]. Genus-level compositional variation further pointed toward evident organ preference among core fungal genera: *Cercospora* prevailed in leaf communities; *Talaromyces*, *Exophiala* and *Fusarium* dominated root mycobiota; floral assemblages were characterised by abundant *Apiotrichum* alongside numerous unclassified fungal lineages. Integrative PCoA, ANOSIM (R = 0.79938, *p* = 0.001) and hierarchical clustering suggested leaf fungal communities may be phylogenetically distinct from the clustered root–stem–flower group, implying leaf tissues impose stronger filtering effects on colonising fungi. LEfSe biomarker profiling further illustrated niche partitioning: roots tended to enrich Eurotiomycetes and Thanatephorus taxa potentially associated with belowground nutrient cycling; stems accumulated differential Diaporthales, *Diaporthe* and *Cyphellophora* genera previously documented to synthesise bioactive secondary metabolites [48,49].

We retrieved 108 culturable isolates from root, stem and leaf tissues, numerically far fewer than the 818 OTUs resolved via amplicon sequencing. Such quantification mismatch commonly occurs in endophyte surveys, where culture-independent sequencing routinely detects substantially higher diversity than culture-based approaches. This discrepancy is often attributed to cultivation bias, as standard laboratory media may not reproduce critical in planta conditions, including host-derived metabolites, oxygen gradients and physicochemical microenvironments required by many uncultured fungi [50].

In comparison with our previous studies on both cultivated *P. crotalarioides* [21] and *Polygala sibirica* [51] from Yunnan, the present study revealed bioactivities in endophytic fungi isolated from wild *P. crotalarioides*. Our earlier study on *P. sibirica* isolated only 23 cultivable strains, of which 12 displayed moderate to high AChE inhibitory activity, with *Fusarium* sp. exhibiting the strongest inhibitory effect. Another previous study isolated 55 endophytic strains from cultivated *P. crotalarioides* and evaluated their AChE inhibitory activity in vitro. Among these, five strains—*Phialophora* sp. PCAM010, *Diaporthe* sp. PCBM027, *Fusarium* sp. LP41, *Fusarium* sp. SR60, and *Phoma* sp. SM81—showed strong activity, with inhibition rates exceeding 50%. By contrast, the present study obtained 108 cultivable fungal strains from wild *P. crotalarioides*, with several strains exhibiting notable dual bioactivities. Specifically, two isolates displayed superior AChE inhibitory activity exceeding 80%, while five strains achieved over 80% DPPH and ABTS radical-scavenging capacity with favorable IC_50_ values. Notably, endophytic fungi isolated from wild *P. crotalarioides* in the present study exhibited higher AChE inhibitory activity than those previously obtained from cultivated *P. crotalarioides* in our prior work. However, these two studies adopted distinct fermentation strategies: the previous study used liquid PDB medium, whereas the current study applied rice-based solid-state fermentation. Accumulating evidence indicates that solid-state fermentation generally yields higher quantities and greater chemical diversity of fungal secondary metabolites than conventional liquid fermentation systems [52,53]. Accordingly, the observed differences in AChE inhibitory activity may be partially explained by disparities in fermentation modes. Nevertheless, several strains isolated from wild *P. crotalarioides* still exhibited prominent AChE inhibitory activity under the unified solid-state fermentation system, suggesting their potential for further investigation. Overall, these bioactivity screening results provide a foundational dataset to support subsequent isolation and purification of bioactive compounds.

Our study revealed that several endophytic fungal strains isolated from wild *P. crotalarioides* exhibited prominent AChE inhibitory and antioxidant activities. These bioactive isolates, primarily assigned to *Penicillium*, *Trichoderma*, *Fusarium*, *Diaporthe*, and *Exserohilum*, represent valuable microbial resources harbored by this subtropical medicinal plant. Although bioactivity evaluations were performed using crude fungal extracts and the observed potency was lower than that of positive controls, the consistent AChE inhibitory and radical-scavenging activities still indicate the metabolic potential of these endophytes. Nevertheless, several limitations should be acknowledged. First, all samples were collected from a single site in Cangyuan County; thus, multi-site sampling across diverse altitudinal and climatic gradients in Yunnan is needed to clarify the geographic effects on endophytic community assembly and functional variation. Second, all bioactivity assessments were limited to in vitro screening of crude extracts; further cellular-level assays and in vivo pharmacological studies are required to validate the practical efficacy and pharmaceutical potential of the prioritized strains. Overall, this work provides foundational strain resources and preliminary bioactivity evidence for future exploration of bioactive metabolites from wild *P. crotalarioides* endophytic fungi.

## 5. Conclusions

In conclusion, wild *Polygala crotalarioides* harbors diverse and tissue-specific endophytic fungal communities, with stems representing the primary reservoir of fungal diversity. Several fungal isolates exhibited promising acetylcholinesterase inhibitory and antioxidant activities, indicating that these strains represent candidates for preliminary bioactivity screening to guide further chemical characterization of active metabolites. These results expand our understanding of plant–fungus associations in this medicinal species and provide a basis for future discovery of bioactive natural products from its endophytic fungal resources.

## Data Availability

Raw sequencing data generated in this study have been deposited in the NCBI BioProject database under accession number PRJNA1207743, with corresponding SRA accessions ranging from SRR31976288 to SRR31976299. Other datasets used and analysed during the current study are available from the authors on reasonable request.

## Supplementary Materials

The following supporting information can be downloaded at https://www.mdpi.com/article/10.3390/jof12090669/s1, Figure S1: Rarefaction curves of fungal communities in different tissues of wild *P. crotalarioides*; The observed species richness (Sobs index) is plotted against the number of sampled reads. R, root; S, stem; L, leaf; F, flower. Figure S2: Stacked bar chart showing the relative abundance of endophytic fungal taxa at the phylum level across four tissue compartments of wild *P. crotalarioides*. R: root; S: stem; L: leaf; F: flower. Taxa with low relative abundance were pooled into the group “others”. Figure S3: Taxonomic composition of culturable endophytic fungi at the phylum level isolated from different tissues of wild *P. crotalarioides*. (A) Root (R); (B) Stem (S); (C) Leaf (L); (D) Flower (F). Each pie chart displays the relative abundance of dominant fungal phyla, including Ascomycota, Basidiomycota, Glomeromycota, and other rare phyla. Table S1: Information of representative isolated fungal strains and yields of their crude fermentation extracts. Table S2: Sequence statistics of endophytic fungi from wild *P. crotalarioides*. Table S3: OTUs of endophytic fungi from wild *P. crotalarioides*. Table S4: ITS Identification Results of Culturable Endophytic Fungi from Wild *P. crotalarioides*. Table S5: Common genera of endophytic fungi in different organs of wild *P. crotalarioides*. Table S6: AChE inhibitory activity and IC_50_ values of endophytic fungi isolated from wild *P. crotalarioides*. Table S7: DPPH radical scavenging activity and IC_50_ values of endophytic fungi isolated from wild *P. crotalarioides*. Table S8: ABTS radical scavenging activity and IC_50_ values of endophytic fungi isolated from wild *P. crotalarioides*.
