# Supplementary material for: Endophytic Fungi from Wild Polygala crotalarioides: Diversity, Antioxidant Capacity and Acetylcholinesterase Inhibitory Potential
Source: J Fungi (Basel). 2026 Sep 6;12(9):669. doi: 10.3390/jof12090669 (PMC13608217; doi:10.3390/jof12090669)
Supplement: Supplementary file 1 [file jof-12-00669-s001.zip › Table S1. Information of representative isolated fungal strains and yields of their crude fermentation extracts.pdf]

Table S1. Information of representative isolated fungal strains and yields of their crude fermentation extracts

Isolates	Closest Relatives in NCBI (Accession No.)	GenBank No.	Extraction yield (%)
PSP020	*Fusarium* sp. (ON003544)	PV176047	10.8
PSP042	*Exserohilum* sp. (MT524320)	PV176056	10.4
PSP053	*Fusarium* sp. (ON950393)	PV176058	12.8
PLM007	*Phomopsis* sp. (MK333965)	PV175972	12.3
PLM009	*Colletotrichum* sp. (MT393840)	PV175973	11.2
PLM013	*Neofusicoccum* sp. (MT261980)	PV175977	8.6
PLM021	*Cercospora* sp. (MH777048)	PV175983	9.6
PRP024	*Penicillium* sp. (MT529099)	PV176022	11.7
PRM002	*Trichoderma* sp. (MT348552)	PV176003	9.2
PRM003	*Diaporthe* sp. (LC477146)	PV176004	8.5
PSM027	*Diaporthe* sp. (KC771480)	PV176039	7.3
PLP001	*Diaporthe* sp. (MT214362)	PV175988	10.5
PLP008	*Cladosporium* sp. (MT223790)	PV175991	8.6
PRM007	*Umbelopsis* sp.. (MT344188)	PV176007	6.9
PRP008	*Trichoderma* sp. (MT102395)	PV176012	7.8
PRP013	*Penicillium* sp. (MK450704)	PV176015	10.2
PRP027	*Didymocyrtis* sp. (LT796877)	PV176023	8.9
PRR009	*Trichoderma* sp. (MH512965)	PV176028	8.2
PSP007	*Colletotrichum* sp. (FJ459934)	PV176041	12.2
PSP100	*Colletotrichum* sp. (MN856443)	PV176070	11.3
PSR009	*Diaporthe* sp. (MH973157)	PV176075	10.2
