# Supplementary material for: Endophytic Fungi from Wild Polygala crotalarioides: Diversity, Antioxidant Capacity and Acetylcholinesterase Inhibitory Potential
Source: J Fungi (Basel). 2026 Sep 6;12(9):669. doi: 10.3390/jof12090669 (PMC13608217; doi:10.3390/jof12090669)
Supplement: Supplementary file 1 [file jof-12-00669-s001.zip › Table S2. Sequence statistics of endophytic fungi from wild P. crotalarioides.pdf]

**Table S2.** Sequence statistics of endophytic fungi from wild *P. crotalarioides*

| Sample name | Number of sequences | Number of bases | Average length | Minimum length | Maximum length |
|---|---|---|---|---|---|
| F1 | 37473 | 10389242 | 277 | 140 | 492 |
| F2 | 38262 | 10661002 | 278 | 140 | 512 |
| F3 | 36700 | 7945144 | 216 | 147 | 523 |
| L1 | 41405 | 10082590 | 243 | 143 | 485 |
| L2 | 48737 | 11135584 | 228 | 140 | 533 |
| L3 | 39557 | 9302039 | 235 | 150 | 533 |
| R1 | 49252 | 12456671 | 252 | 158 | 481 |
| R2 | 31294 | 7996008 | 255 | 156 | 505 |
| R3 | 48197 | 10703356 | 222 | 172 | 507 |
| S1 | 55888 | 13715040 | 245 | 140 | 528 |
| S2 | 59876 | 14453905 | 241 | 147 | 538 |
| S3 | 51887 | 12459854 | 240 | 140 | 533 |
