# Supplementary material for: Endophytic Fungi from Wild Polygala crotalarioides: Diversity, Antioxidant Capacity and Acetylcholinesterase Inhibitory Potential
Source: J Fungi (Basel). 2026 Sep 6;12(9):669. doi: 10.3390/jof12090669 (PMC13608217; doi:10.3390/jof12090669)
Supplement: Supplementary file 1 [file jof-12-00669-s001.zip › Table S5. Common genera of endophytic fungi in different organs of wild P. crotalarioides.pdf]

Table S5. Common genera of endophytic fungi in different organs of wild *P. crotalarioides*

Sample groups	Number of shared genera	Shared genus names
Leaf-Root-Stem	4	*Colletotrichum*<br>*Diaporthe*<br>*Fusarium*<br>*Penicillium*
Leaf-Root	1	*Trichoderma*
Leaf-Stem	3	*Cercospora*<br>*Exserohilum*<br>*Neofusicoccum*
