# Supplementary material for: Endophytic Fungi from Wild Polygala crotalarioides: Diversity, Antioxidant Capacity and Acetylcholinesterase Inhibitory Potential
Source: J Fungi (Basel). 2026 Sep 6;12(9):669. doi: 10.3390/jof12090669 (PMC13608217; doi:10.3390/jof12090669)
Supplement: Supplementary file 1 [file jof-12-00669-s001.zip › Table S6. AChE inhibitory activity and IC50 values of endophytic fungi isolated from wild P. crotalarioides..pdf]

Table S6. AChE inhibitory activity and IC_{50} values of endophytic fungi isolated from wild *P. crotalarioides*.

Isolates	Closest Relatives in NCBI (Accession No.)	GenBank No.	AChE Inhibition%	IC_{50} (μg/mL)	IC_{50} Range (μg/mL)
PLM021	*Cercospora* sp. (MH777048)	PV175983	73.51±0.97*	418.1	393.6 ~ 449.6
PLM009	*Colletotrichum* sp. (MT393840)	PV175973	43.85±0.4	NA	NA
PSP007	*Colletotrichum* sp. (FJ459934)	PV176041	27.22±0.91	NA	NA
PSP100	*Colletotrichum* sp. (MN856443)	PV176070	56.41±1.30*	710.6	685.2 ~ 737.3
PLP001	*Diaporthe* sp. (MT214362)	PV175988	44.79±0.7	NA	NA
PSR009	*Diaporthe* sp. (MH973157)	PV176075	53.77±1.83*	827.5	774.6 ~ 882.3
PRP027	*Didymocyrtis* sp. (LT796877)	PV176023	39.31±0.8	NA	NA
PSP042	*Exserohilum* sp. (MT524320)	PV176056	71.11±0.35*	660.0	601.8 ~ 719.2
PSP020	*Fusarium* sp. (ON003544)	PV176047	36.21±0.77	NA	NA
PSP053	*Fusarium* sp. (ON950393)	PV176058	75.58±0.81*	566.4	545.5 ~ 588.9
PLM013	*Neofusicoccum* sp. (MT261980)	PV175977	45.12±0.74	NA	NA
PRP013	*Penicillium* sp. (MK450704)	PV176015	34.8±1.73	NA	NA
PRP024	*Penicillium* sp. (MT529099)	PV176022	83.87±1.51**	536.7	536.7
PLM007	*Phomopsis* sp. (MK333965)	PV175972	28.72±1.05	NA	NA
PRM002	*Trichoderma* sp. (MT348552)	PV176003	25.85±1.38	NA	NA
PRP008	*Trichoderma* sp. (MT102395)	PV176012	82.53±0.80**	441.3	441.9 ~ 470.2
PRR009	*Trichoderma* sp. (MH512965)	PV176028	22.55±1.48	NA	NA
PRM007	*Umbelopsis* sp. (MT344188)	PV176007	29.53±1.25	NA	NA

Note: Values are presented as mean ± standard deviation (SD, n=3) at a concentration of 2000 μg/mL. Galanthamine was used as the positive control, with an inhibition rate of 98.3 ± 0.2%, an IC_{50} value of 67.5 μg/mL, and an IC_{50} range (95% confidence interval) of 64.00–71.08 μg/mL. * indicates strains with AChE inhibition > 50%, and ** indicates strains with inhibition > 80%. The IC_{50} values were calculated for strains with inhibition ≥ 50% at the tested concentration; NA indicates that IC_{50} was not determined (inhibition < 50% at 2000 μg/mL).
