# Supplementary material for: Endophytic Fungi from Wild Polygala crotalarioides: Diversity, Antioxidant Capacity and Acetylcholinesterase Inhibitory Potential
Source: J Fungi (Basel). 2026 Sep 6;12(9):669. doi: 10.3390/jof12090669 (PMC13608217; doi:10.3390/jof12090669)
Supplement: Supplementary file 1 [file jof-12-00669-s001.zip › Table S7. DPPH radical scavenging activity and IC50 values of endophytic fungi isolated from wild P. crotalarioides.pdf]

Table S7. DPPH radical scavenging activity and IC_{50} values of endophytic fungi isolated from wild *P. crotalarioides.*

Isolates	Closest Relatives in NCBI (Accession No.)	GenBank No.	DPPH radical scavenging activity (%)	IC_{50} (μg/mL)	IC_{50} Range (μg/mL)
PLM021	*Cercospora* sp. (MH777048)	PV175983	94.25±1.44**	771.5	745.6 ~ 797.4
PSM027	*Diaporthe* sp. (KC771480)	PV176039	95.67±1.92**	725.6	683.1 ~ 771.4
PRM003	*Diaporthe* sp. (LC477146)	PV176004	87.75±1.86*	717.0	657.6 ~ 779.7
PSP042	*Exserohilum* sp. (MT524320)	PV176056	84.76±2.00*	258.7	238.8 ~ 275.0
PSP020	*Fusarium* sp. (ON003544)	PV176047	93.38±1.14*	466.6	414.1 ~ 514.7
PSP053	*Fusarium* sp. (ON950393)	PV176058	98.03±1.19**	436.0	388.4 ~ 487.7
PLM013	*Neofusicoccum* sp. (MT261980)	PV175977	91.99±1.30*	693.6	649.8 ~ 742.3

Note: Values are presented as mean ± standard deviation (SD, n=3) at a concentration of 2000 μg/mL. Vitamin C was used as the positive control, with a scavenging activity of 97.36 ± 0.59%, an IC_{50} value of 10.10 μg/mL, and an IC_{50} range (95% confidence interval) of 8.20–12.36 μg/mL. * indicates strains with DPPH radical scavenging activity > 80%, and ** indicates strains with activity > 90%. IC_{50} values were calculated for strains with scavenging activity ≥ 50% at the tested concentration, with the IC_{50} range representing the 95% confidence interval (μg/mL).
