# Supplementary material for: Endophytic Fungi from Wild Polygala crotalarioides: Diversity, Antioxidant Capacity and Acetylcholinesterase Inhibitory Potential
Source: J Fungi (Basel). 2026 Sep 6;12(9):669. doi: 10.3390/jof12090669 (PMC13608217; doi:10.3390/jof12090669)
Supplement: Supplementary file 1 [file jof-12-00669-s001.zip › Table S8. ABTS radical scavenging activity and IC50 values of endophytic fungi isolated from wild P. crotalarioides.pdf]

Table S8. ABTS radical scavenging activity and IC_{50} values of endophytic fungi isolated from wild *P. crotalarioides*.

Isolates	Closest Relatives in NCBI (Accession No.)	GenBank No.	ABTS radical scavenging activity (%)	IC_{50} (μg/mL)	IC_{50} Range (μg/mL)
PLP008	*Cladosporium* sp. (MT223790)	PV175991	90.63±0.84**	695.2	663.6 ~ 728.0
PLM009	*Colletotrichum* sp. (MT393840)	PV175973	85.80±1.34*	712.5	676.4 ~ 749.8
PSM027	*Diaporthe* sp. (KC771480)	PV176039	95.52±1.89**	640.4	622.6 ~ 658.5
PRM003	*Diaporthe* sp. (LC477146)	PV176004	87.31±1.74*	656.7	595.8 ~ 717.7
PSP042	*Exserohilum* sp. (MT524320)	PV176056	84.19±1.97*	692.4	675.8 ~ 708.5
PSP020	*Fusarium* sp. (ON003544)	PV176047	93.10±1.22**	585.7	567.9 ~ 603.1
PSP053	*Fusarium* sp. (ON950393)	PV176058	90.43±1.51**	418.7	402.4 ~ 434.7
PRP024	*Penicillium* sp. (MT529099)	PV176022	87.56±0.83*	423.2	402.6 ~ 443.6
PRM002	*Trichoderma* sp. (MT348552)	PV176003	82.63±1.05*	664.3	627.1 ~ 701.1

Note: Values are presented as mean ± standard deviation (SD, n=3) at a concentration of 2000 μg/mL. Vitamin C was used as the positive control, with a scavenging activity of 97.23 ± 0.63%, an IC_{50} value of 70.06 μg/mL, and an IC_{50} range (95% confidence interval) of 42.16–83.80 μg/mL. * indicates strains with ABTS radical scavenging activity > 80%, and ** indicates strains with activity > 90%. IC_{50} values were calculated for strains with scavenging activity ≥ 50% at the tested concentration, with the IC_{50} range representing the 95% confidence interval (μg/mL).
