# Supplementary material for: Endophytic Fungi from Wild Polygala crotalarioides: Diversity, Antioxidant Capacity and Acetylcholinesterase Inhibitory Potential
Source: J Fungi (Basel). 2026 Sep 6;12(9):669. doi: 10.3390/jof12090669 (PMC13608217; doi:10.3390/jof12090669)
Supplement: Supplementary file 1 [file jof-12-00669-s001.zip › Figure S1. Rarefaction curves of fungal communities in different tissues of wild P. crotalarioides.pdf]

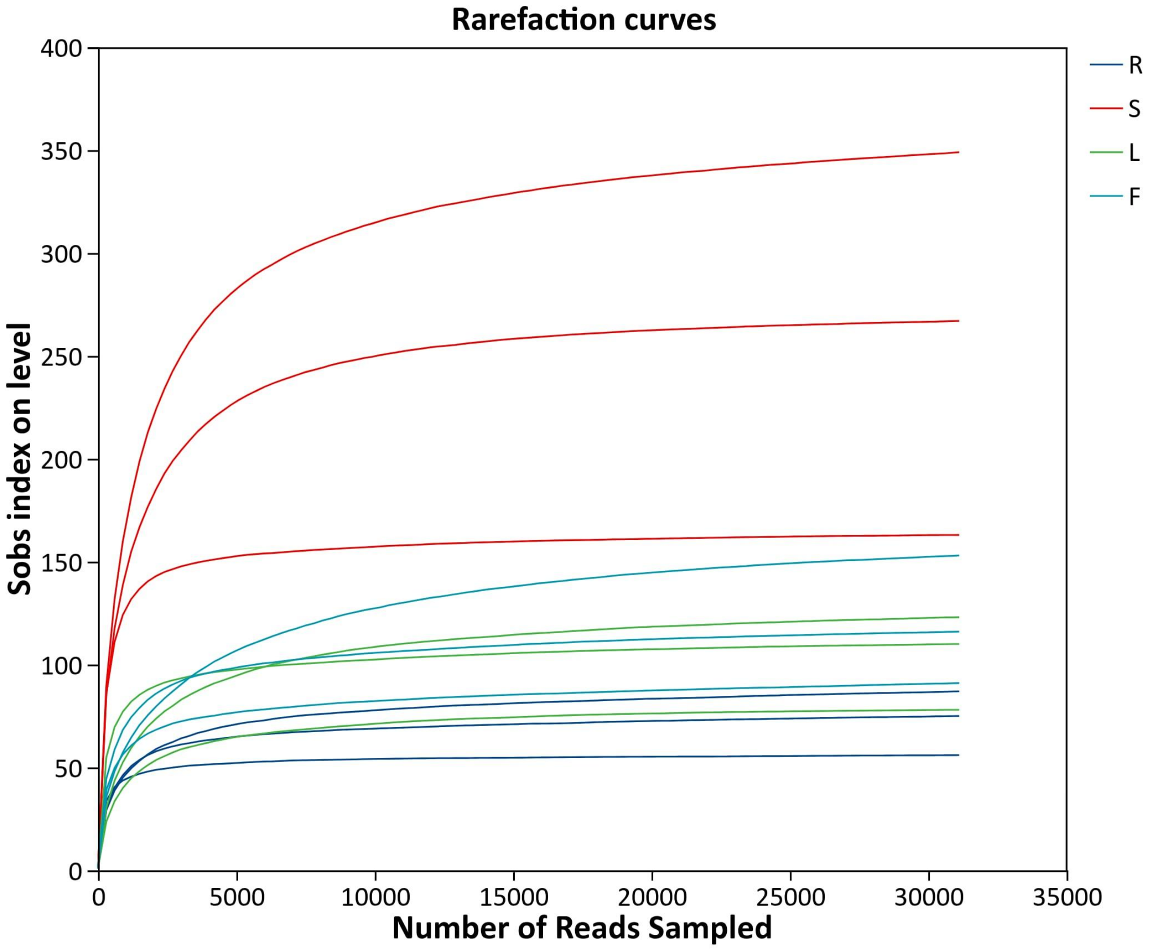

Figure. S1. Rarefaction curves of fungal communities in different tissues of wild *P. crotalarioides*. The observed species richness (Sobs index) is plotted against the number of sampled reads. R, root; S, stem; L,leaf; F, flower.
