# Supplementary material for: Endophytic Fungi from Wild Polygala crotalarioides: Diversity, Antioxidant Capacity and Acetylcholinesterase Inhibitory Potential
Source: J Fungi (Basel). 2026 Sep 6;12(9):669. doi: 10.3390/jof12090669 (PMC13608217; doi:10.3390/jof12090669)
Supplement: Supplementary file 1 [file jof-12-00669-s001.zip › Figure S2. Stacked bar chart showing the relative abundance of endophytic fungal taxa atthephylum level across four tissue compartments of wild P. crotalarioides.pdf]

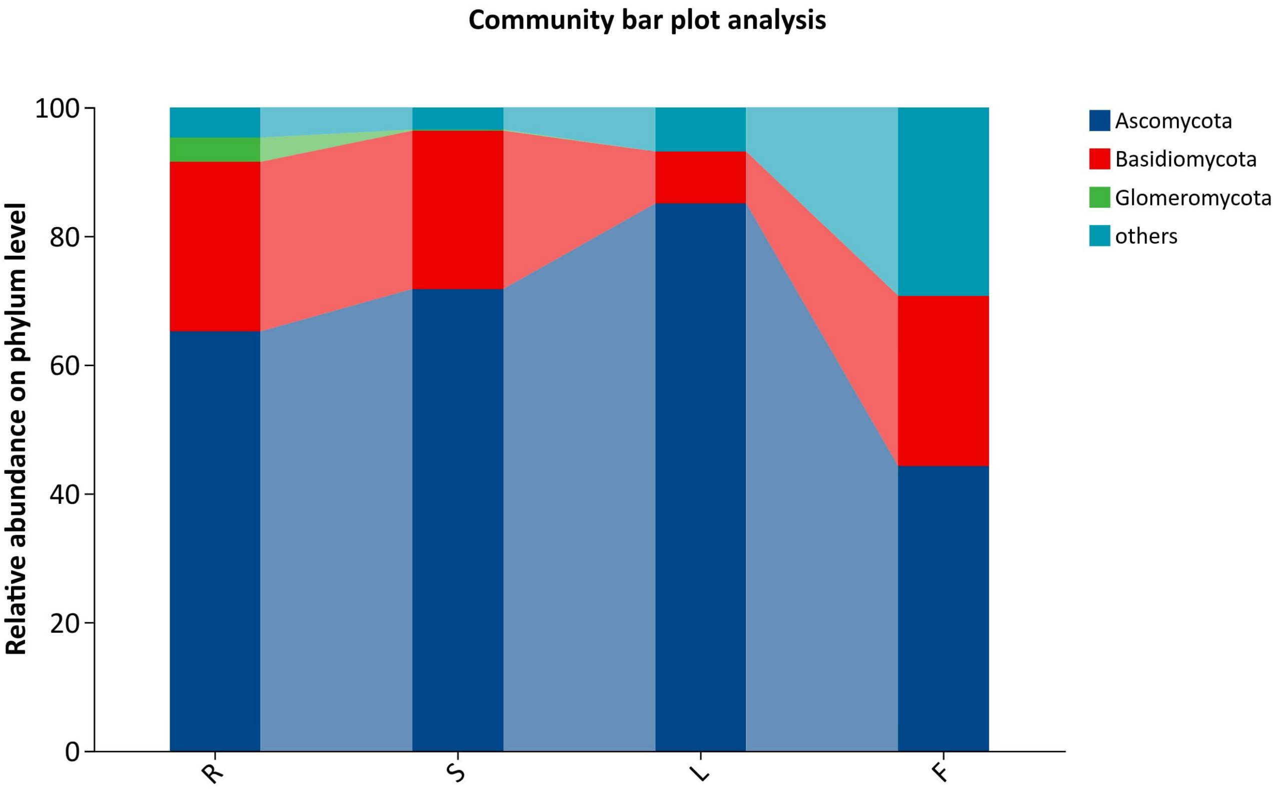

Figure. S2. Stacked bar chart showing the relative abundance of endophytic fungal taxa at the phylum level across four tissue compartments of wild *P. crotalarioides*. R: root; S: stem; L: leaf; F: flower. Taxa with low relative abundance were pooled into the group “others”.
