# Supplementary material for: Endophytic Fungi from Wild Polygala crotalarioides: Diversity, Antioxidant Capacity and Acetylcholinesterase Inhibitory Potential
Source: J Fungi (Basel). 2026 Sep 6;12(9):669. doi: 10.3390/jof12090669 (PMC13608217; doi:10.3390/jof12090669)
Supplement: Supplementary file 1 [file jof-12-00669-s001.zip › Figure S3 Taxonomic composition at the phylum level of culturable endophytic fungi isolated from different tissues of wild Polygala crotalarioides .pdf]

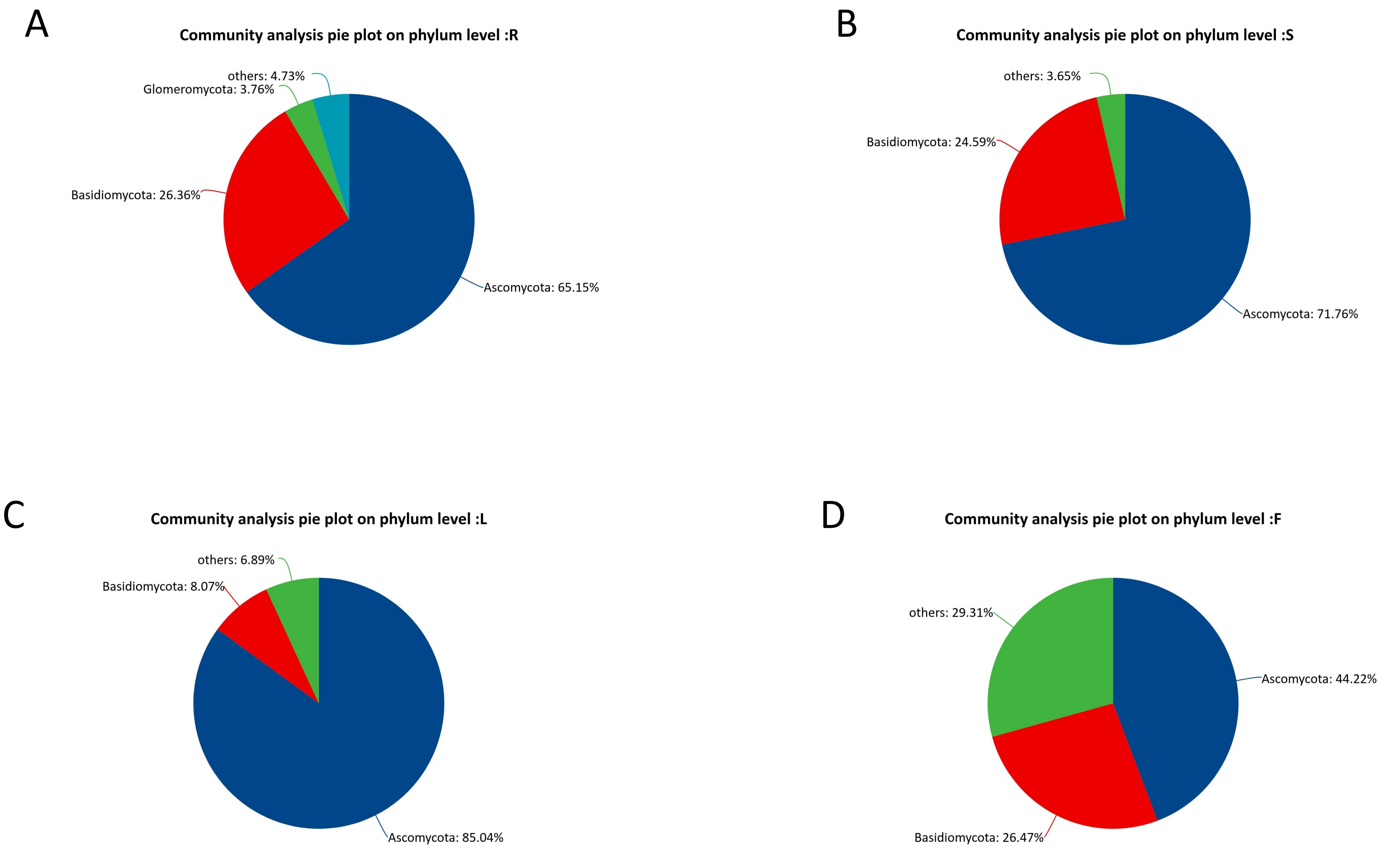

Fig. S3. Taxonomic composition of culturable endophytic fungi at the phylum level isolated from different tissues of wild *P. crotalarioides*. (A) Root (R); (B) Stem (S); (C) Leaf (L); (D) Flower (F). Each pie chart displays the relative abundance of dominant fungal phyla, including Ascomycota, Basidiomycota, Glomeromycota, and other rare phyla.
